# Organic Transistor‐Based Neuromorphic Electronics and Their Recent Applications

**DOI:** 10.1002/smtd.202501966

**Published:** 2026-01-24

**Authors:** Ziru Wang, Feng Yan

**Affiliations:** ^1^ Department of Applied Physics Research Centre for Organic Electronics The Hong Kong Polytechnic University Kowloon Hong Kong P. R. China; ^2^ Department of Materials Science and Engineering Southern University of Science and Technology Shenzhen P. R. China

**Keywords:** hardware computing, organic neuromorphic electronics, organic transistors

## Abstract

Neuromorphic technologies offer a promising pathway to address the escalating energy demands of artificial intelligence. At the system level, neuromorphic computing seeks to overcome the von Neumann bottleneck by integrating memory and processing, while neuromorphic sensing minimizes redundant data transfer by processing signals directly at the point of acquisition. Organic transistors have emerged as compelling candidates for emulating synaptic and neuronal behaviors owing to their low power consumption, flexibility, stretchability, and biocompatibility, making them particularly attractive for bio‐related neuromorphic applications. This review provides an overview of organic transistor‐based artificial synapses and neurons, with emphasis on the mechanisms underlying their neuromorphic behaviors. Subsequently, recent advances in applications, broadly categorized into neuromorphic computing and neuromorphic sensing, are summarized and representative bio‐integrated demonstrations are highlighted. Finally, we outline key challenges at the material, device, and system levels, and discuss future opportunities for advancing organic neuromorphic electronics toward practical, biocompatible, and intelligent systems.

## Graphical abstract

This review highlights recent progress in organic neuromorphic electronics, showing how organic semiconductors enable synaptic and neuronal functions with low power, mechanical flexibility, and biocompatibility. By bridging materials, devices, and systems, organic platforms are accelerating brain‐inspired computing toward applications in artificial intelligence, robotics, neuroprosthetics, and wearable bioelectronics.

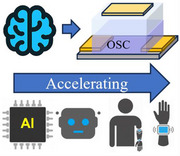

## 1 Introduction

The conventional von Neumann computing architecture based on physically separated processing and memory units has made unparalleled contributions to science and technology over the past decades. However, in emerging artificial intelligence (AI) applications, the ever‐growing demand for processing massive datasets and increasingly complex models has exposed a critical bottleneck: the time and energy cost of shuttling data between memory and processor is becoming unsustainable [1, 2, 3, 4, 5]. In contrast to modern computers, the human brain, comprising approximately 10^11^ neurons and 10^15^ synapses [6], can process complex and unstructured information with remarkable speed, while maintaining an ultra‐low energy consumption of ∼20 W [7, 8, 9]. Motivated by the intelligence and efficiency of biological neural systems, brain‐inspired computing utilizing artificial neurons and synapses has intrigued substantial research interests for its potential in achieving massive parallelism and superior energy efficiency beyond traditional silicon‐based computers.

The hardware implementation of neuronal and synaptic functionalities forms the foundation of neuromorphic computing, and over the past several decades substantial progress has been made in this area, enabling the development of neuromorphic systems based on artificial neurons and synapses [10]. In 1990, Carver Mead introduced the term “neuromorphic electronic system” to describe the devices and circuits which emulate the basic operations of the biological nervous system [11]. On one hand, silicon‐based neuromorphic circuits have been developed to reproduce spiking neuron behaviors [12, 13, 14, 15], and large‐scale silicon spiking neural networks have been realized in neuro‐inspired computing platforms such as SpiNNaker from University of Manchester [16], Neurogrid from Stanford University [17], IBM's TrueNorth [18], and Intel's Loihi [19, 20], achieving orders of magnitude improvements in energy efficiency per event. However, these CMOS‐based neuromorphic circuits typically require more than ten transistors to emulate the function of a single synapse or neuron, resulting in high design complexity, more energy cost and presenting significant challenges for further large‐scale integration in the post‐Moore's law era [21, 22, 23].

On the other hand, ever since the initial experimental demonstration of the memristor [24], emerging memristive devices (the term “memristor” is avoided here, as its original definition is not always fully applicable) [8, 25] have garnered considerable attention as promising candidates for neuromorphic applications. By encoding the tunable plasticity of biological synapses in multiple electronic resistance states, these devices can mimic essential synaptic behaviors within a single electronic component, thereby providing a more compact and energy‐efficient alternative to conventional CMOS‐based circuits.

Through the years, a broad range of novel functional materials has been developed to emulate synaptic functions, including phase‐change materials [26, 27], metal oxides [28, 29, 30, 31], magnetic materials [32], 2D materials [33, 34, 35], and organic materials [36, 37, 38, 39, 40]. Among these, organic neuromorphic electronics have attracted particular attention due to their favorable performance metrics in neuromorphic applications involving low energy consumption, high cycling endurance, fast switching speed and sufficient retention time, as well as unique properties of synthetic tunability, mechanical flexibility, stretchability and biocompatibility [8, 41, 42, 43, 44, 45]. These attributes not only enable organic neuromorphic electronics to reproduce fundamental neuromorphic behaviors, such as short‐term plasticity and long‐term plasticity for artificial synapses, and signal integration and an excitation threshold for artificial neurons but also position them as strong candidates for advanced neuromorphic technologies, particularly those involving bio‐signal processing or operation within biological environment.

In this review, we provide an overview of recent advances in organic neuromorphic electronics across both device and circuit levels, as well as their emerging applications. We begin with a brief introduction to biological synapses and neuronal spiking behaviors, establishing the biological principles which inspire device and circuit design. We then introduce the principal organic transistor structures and review the key state‐switching mechanisms underlying transistor‐based organic artificial synapses, focusing on their ability to emulate short‐ and long‐term plasticity in response to conventional electrical gating and diverse external stimuli, including optical, mechanical, chemical, and biochemical inputs. Section 5 highlights recent progress in organic artificial neurons, with emphasis on circuit design innovations and performance improvements. Section 6 discusses representative neuromorphic applications enabled by organic devices, encompassing neuromorphic computing and neuromorphic sensing, with particular attention to bio‐integrated systems. Finally, we conclude with a summary of the field, outlining the remaining challenges and identifying promising future directions for organic neuromorphic electronics.

## 2 Biological Inspirations: Synaptic Functions and Spiking Behaviors

### 2.1 Biological Synaptic Functions

**FIGURE 1:**
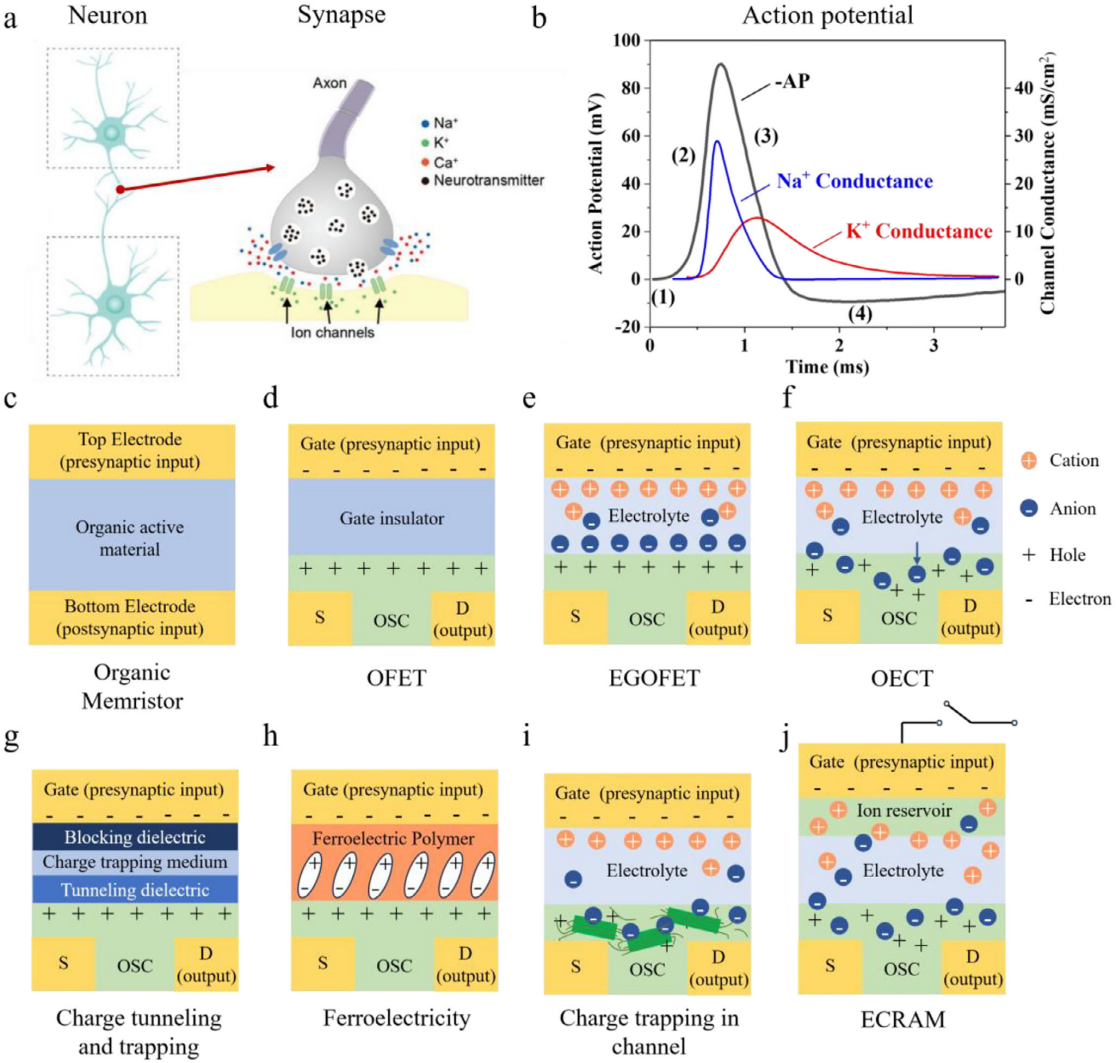
Schematics of a biological chemical synapse. Adapted with permission [53]. Copyright 2019, WILEY‐VCH. b) AP waveform (black) with corresponding Na^+^ channel (blue) and K^+^ channel (red) conductance profiles. The AP is segmented into four phases: (1) resting state, (2) depolarization, (3) repolarization, and (4) hyperpolarization. Adapted with permission [54]. Copyright 1952, The Physiological Society. Typical device structure of c) memristor‐based organic artificial synapses, d) OEFT, e) EGOFET, f) OECT, g) OFET employing charge‐tunneling/trapping, h) OFET with a ferroelectric dielectric layer, i) OECT with channel charge trapping, and j) ECRAM. S: source electrode; D: drain electrode; OSC: organic semiconductor.

Synapses, served as the basic unit of brain, reside in the interneuronal cellular junctions and are responsible for transmitting signals between neurons [46]. Figure 1a illustrates the schematic of a chemical synapse, in which information is transmitted via the release of neurotransmitters from the presynaptic terminal to the postsynaptic terminal. In contrast, electrical synapses employ gap junctions which directly connect the cytoplasm of adjacent cells, enabling bidirectional transmission of electrical signals [47]. The postsynaptic response can vary substantially depending on the synaptic strength through which the presynaptic signals propagate.

Regulation of synaptic strength can be broadly classified into short‐term plasticity (STP) and long‐term plasticity (LTP) based on the duration of the induced changes. STP, which typically lasts from milliseconds to minutes, enables synapse to perform crucial computational functions in neural circuits such as synaptic filtering, adaptive responding, dynamic depression, and synaptic input interaction [48]. Typical STP activities include short‐term facilitation, short‐term depression and pair‐pulsed facilitation/depression [49]. LTP, where the synaptic strength modification persists for hours or longer, plays a pivotal role in learning and memory formation [50]. For instance, through Hebbian learning principles, memory formation can occur accompanied by lasting changes in synaptic efficiency [51, 52].

### 2.2 Biological Spiking Behaviors

In the biological nervous system, action potentials (APs), or spikes, are highly stereotyped electrical signals through which the brain receives, processes, and transmits information [55]. These signals—initiated by a wide variety of events, such as sensory transduction in visual or olfactory neurons—are identical by the form, regardless of their origin. The molecular basis of all electrical activity in the nervous system lies in ion channels, which can be broadly classified into voltage‐gated ion channels (VGICs) and ligand‐gated ion channels (LGICs) [53].

The gating mechanisms of VGICs are fundamental to the initiation and propagation of APs in excitable cells [53]. The process can be summarized as follows (Figure 1b): at rest, the Na^+^‐K^+^ pumps actively transport Na^+^ out of the cell and K^+^ into the cell, while the membrane maintains selective permeability to K^+^, thereby sustaining a negative resting membrane potential. When a stimulus depolarizes the membrane beyond a threshold, voltage‐gated sodium (Nav) channels open rapidly, rendering the membrane more permeable to Na^+^ than to K^+^. The resultant influx of Na^+^ further depolarizes the membrane and enhances Na^+^ permeability, creating a positive feedback loop that accelerates depolarization. This depolarization also activates voltage‐gated potassium (Kv) channels, leading to a K^+^ efflux. As depolarization persists, Nav channels become inactivated, whereas Kv channels remain open as long as the membrane remains depolarized, restoring the membrane potential back toward its resting value, producing repolarization and often a subsequent transient hyperpolarization [53, 55, 56, 57]. Notably, the AP operates on an all‐or‐none principle: stimuli below the threshold fail to generate a signal, whereas stimuli above the threshold all produce APs of uniform amplitude [55].

## 3 Device Fundamentals: Organic Transistors for Neuromorphic Systems

To emulate synaptic signal transmission or neuronal spiking behaviors, researchers have developed organic neuromorphic electronics in both two‐terminal (2T) memristor‐based and three‐terminal (3T) transistor‐based configurations, each offering tunable conductance states [9, 43, 58, 59]. A typical 2T organic memristive device consists of an organic active layer sandwiched between two metal electrodes (Figure 1c), whereas a 3T organic neuromorphic device comprises source, drain and gate electrodes. In the latter, an organic (semi‐)conducting thin film is positioned between the source and drain electrode as the channel, with the gate electrode coupled through a dielectric layer or electrolyte [8]. Organic transistor‐based neuromorphic devices are generally classified into two main categories: organic field‐effect transistors (OFETs) and organic electrochemical transistors (OECTs), distinguished by their respective operating principles. These two device types constitute the primary focus of this review, whereas detailed discussions on neuromorphic devices based on 2T configurations can be found in other comprehensive reviews [8, 60, 61].

### 3.1 OFET

An OFET adopts the thin‐film transistor architecture, where the semiconducting organic channel is separated from the gate electrode by a solid‐state dielectric (Figure 1d). Application of a gate voltage induces the accumulation of charge carriers at the channel/dielectric interface via the field effect doping, thereby modulating the channel current [62]. A widely studied subclass, the electrolyte‐gated OFET (EGOFET), replaces the solid dielectric with an ionically conductive but electronically insulating electrolyte (Figure 1e) [9]. In this case, gate modulation is mediated by the formation of ultra‐thin electrical double layers (EDLs) at the electrolyte/semiconductor and electrolyte/gate interfaces. This yields a greatly enhanced interfacial capacitance, enabling EGOFETs to operate at low voltages while preserving the general field‐effect mechanism.

### 3.2 OECT

OECTs share a similar electrolyte‐gated architecture but operate on a fundamentally different principle (Figure 1f) [41]. Instead of interfacial field‐effect doping, OECTs rely on bulk electrochemical doping and dedoping of an ion‐permeable semiconducting channel. When a gate bias is applied, ions from the electrolyte are injected into the channel, altering its oxidation state and thereby tuning the electronic conductivity. This volumetric doping mechanism endows OECTs with exceptionally high transconductance at low operating voltages, and their mixed ionic–electronic conduction provides a striking analogy to biological neural signaling [40]. These properties have made OECTs the most prominent candidates among organic transistors for neuromorphic applications.

Building on these fundamental device architectures, a diverse range of organic neuromorphic electronic systems—spanning individual devices to integrated circuits—has been demonstrated. Their development and applications are reviewed in detail in the following sections.

## 4 Organic Artificial Synapse

Emulating the functional characteristics of biological synapses, particularly variation of synaptic plasticity (synaptic strength), is a fundamental requirement for constructing artificial synapses. In a 2T synaptic device, operation typically involves applying a presynaptic signal (write voltage) to one electrode, followed by measuring the resulting postsynaptic response (current) under a separate read voltage [58]. In contrast, a 3T neuromorphic device applies the presynaptic signal to a gate electrode, while the postsynaptic response—manifested as changes in the conductivity of the semiconducting channel—is measured via a voltage applied between the source and drain electrodes, thus enabling physically separated input and output pathways (Figure 1d–f) [21, 63].

The decoupling of the “read” and “write” pathways has led to growing interest in 3T organic artificial synapses, owing to their operational stability, capacity for multimodal and synergistic control, and improved state retention and energy efficiency [9, 45]. These advantages endow 3T organic artificial synapses with significant potential for the realization of robust and multifunctional neuromorphic systems. Accordingly, this section focuses on the conductance‐tuning mechanisms that have been explored in 3T organic synaptic devices for emulating both STP and LTP, primarily charge trapping (Figure 1g,i), dipole alignment (Figure 1h), and electrochemical switching (Figure 1f,j).

### 4.1 Synaptic Function Mechanisms in Organic Transistors

#### 4.1.1 Charge Trapping Switching

**FIGURE 2:**
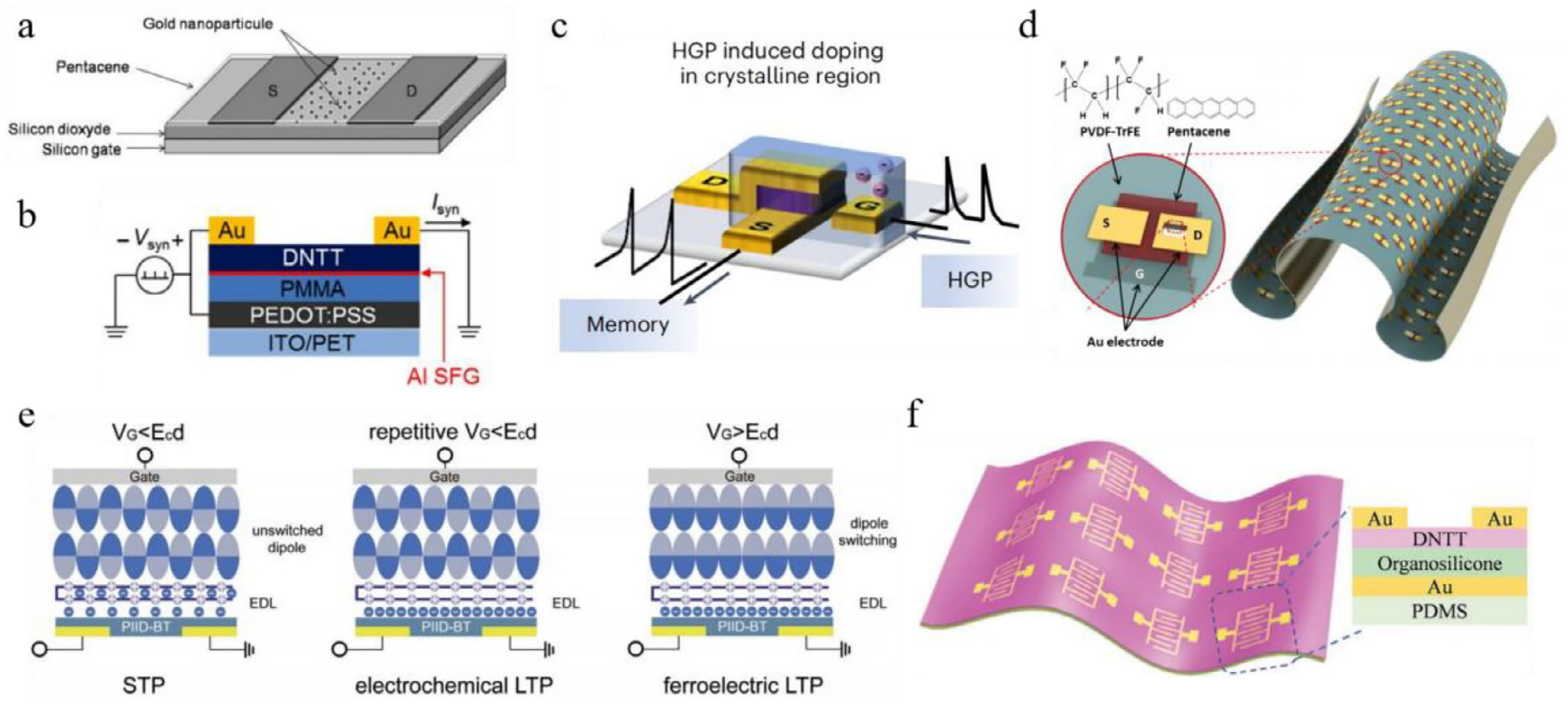
a) Gold NOMFET with charge trapping in a pentacene channel. Reproduced with permission [65]. Copyright 2010, WILEY‐VCH. b) Structure of a DNTT SFG‐OFET showing an ultrathin Al self‐formed floating‐gate layer formed between DNTT and PMMA. Reproduced with permission [70]. Copyright 2016, Springer Nature. c) v‐OECT functioning as a non‐volatile synapse. Reproduced with permission [74]. Copyright 2023, Springer Nature. d) Ultrathin conformable freestanding FONTs and the molecular structure of P(VDF‐TrFE) and pentacene. Reproduced with permission [76]. Copyright 2019, American Chemical Society. e) Ferroelectric/electrochemical synaptic device structure and its operating mechanisms: STP, electrochemical‐doping‐induced LTP, and ferroelectricity‐induced LTP. Reproduced with permission [78]. Copyright 2018, WILEY‐VCH. f) Flexible synaptic transistor with DNTT channel, organosilicone dielectric, and PDMS elastomer substrate. Reproduced with permission [79]. Copyright 2019, WILEY‐VCH.

Charge trapping is a widely adopted memory mechanism for achieving both STP and LTP in OFETs. Charge traps can be embedded within the organic semiconductor or located in the gate dielectric [8]. Vuillaume et al. developed an OFET incorporating a pentacene active layer with a monolayer of gold nanoparticles immobilized at the pentacene/dielectric interface (NOMFET), achieving a retention time of up to 4500 s [64]. Using the same device architecture (Figure 2a), they subsequently demonstrated STP responses to spiking inputs [65]. In this configuration, the interfacial nanoparticles acted as nanoscale capacitors that stored electrical charges, which in turn electrostatically repelled holes in the pentacene channel, thereby modulating its conductance. STP responses were also demonstrated in an EGOFET with Au nanoparticles embedded in the pentacene semiconducting layer [66]. Besides Au nanoparticles, black phosphorus‐ZnO hybrid nanoparticles have also been employed as the charge trapping layer between a SiO_2_ dielectric and a pentacene semiconductor of an OFET, enabling the transistor to emulate multiple long‐term synaptic functions and realize four discrete synaptic weights [67].

Floating‐gated OFETs, in which charge trapping occurs within the dielectric layer, have been extensively studied for non‐volatile memory applications [68, 69]. The gate dielectric in these devices typically consists of three functional layers: a tunneling dielectric layer, a charge‐trapping layer, and a blocking dielectric layer (Figure 1g). Under an applied gate bias, charges from the semiconducting channel are injected through the tunneling layer and stored in the charge‐trapping layer, while the blocking layer prevents further charge transfer to or from the gate electrode. More recently, floating‐gated OFETs have also been explored for emulating synaptic functions. For instance, Kim and colleagues reported an organic synaptic device employing dinaphtho[2,3‐b:2′,3′‐f] thieno[3,2‐b]thiophene (DNTT) as the active channel and a vacuum‐deposited ultra‐thin aluminum nanosheet as the charge trapping layer (Figure 2b) [70]. In this architecture, PMMA and the naturally formed aluminum oxide (Al_2_O_3_) functioned as the blocking and tunneling dielectric layers, respectively. The resulting self‐formed floating‐gate OFET (SFG‐OFET) successfully reproduced STP observed in biological synapses. Similarly, Zhou et al. demonstrated an organic floating gate transistor incorporating fullerene (C_60_) nanoparticles as trapping sites within the tunneling PMMA dielectric layer [71]. This C_60_/PMMA hybrid dielectric layer not only reduced the operating voltage to 4/‐8 V but also enhanced device stability, enabling over 500 programming/erasing cycles. Both STP and LTP were achieved in this molecular hybrid dielectric synaptic transistor. In another example, blending PVN and N2200 as a hybrid trapping layer between a SiO_2_ dielectric and a J71 semiconductor yielded an OFET capable of LTP persisting for several tens of seconds [72].

Charge trapping mechanisms have also been employed to realize non‐volatile synaptic behaviors in OECTs through microstructure engineering (Figure 1i) [73, 74, 75]. In a recent study, Wang et al. developed a dual‐mode OECT with a vertical transverse architecture (v‐OECT) capable of multi‐modal sensing and non‐volatile memory [74]. The v‐OECT incorporated a crystalline‐amorphous organic mixed ionic‐electronic conductor (OMIEC) channel based on PTBT‐p thin films annealed at 200°C, gated by 1‐ethyl‐3‐methylimidazolium bis(trifluoromethylsulfonyl)imide (EMIM:TFSI): poly(vinylidene fluoride‐*co*‐hexafluoropropylene) (PVDF‐HFP) ion gel and polarizable gold electrodes (Figure 2c). This device exhibited STP under low gate doping potential (LGP) and LTP under high gate doping potential (HGP). Experimental analysis revealed that LGP doped the amorphous regions of the channel in a volatile manner, whereas HGP induced anion trapping among the ordered and compact glycol side chains or blockage by the bulky crystalline domains, with de‐trapping occurring only under sufficiently high positive potentials. As a result, the v‐OECT achieved long‐term retention exceeding 10,000 s and supported 1,024 (10‐bit) distinct conductance states with the gate grounded in ambient air.

#### 4.1.2 Dipole Alignment Switching

Dipole alignment, most prominently ferroelectricity, constitutes another key memory mechanism for constructing organic synaptic devices. In such systems, the ferroelectric polarization, modulated by the applied gate voltage, alters the charge carrier concentration in the channel material, thereby tuning its conductance (Figure 1h). For instance, Kim and co‐workers reported an ultrathin conformable freestanding ferroelectric organic neuromorphic transistor (FONT) utilizing poly(vinylidenedifluoride)‐trifluoroethylene P(VDF‐TrFE) as both the gate dielectric and the supporting layer (Figure 2d) [76]. By controlling the remnant polarization of the organic ferroelectric film P(VDF‐TrFE) through gate voltage, the charge carrier concentration in the pentacene channel was effectively modulated. Through precise adjustment of the gate‐spike intensity and duration, the device was able to emulate both short‐term and long‐term synaptic plasticity. More recently, a non‐ferroelectric poly(N‐vinylcarba‐zole) (PVK) passivation layer was introduced between the P(VDF‐TrFE) ferroelectric film and the organic semiconductor poly‐[2,5‐bis(2‐octyldodecyl)‐3,6‐di(thiophen‐2‐yl)pyrrolo[3,4‐c]pyrrole‐1,4(2H,5H)‐dionel‐alt‐thieno[3,2‐b]thiophene] (DPPT‐TT) to reduce dielectric/semiconductor interface roughness and suppress gate leakage [77]. At a PVK film thickness of 19 nm, this device exhibited optimized ferroelectric properties with minimized gate leakage, attributed to the PVK's role as an effective charge barrier and its suppression of interfacial trap formation. This Fe‐OFET demonstrated excitatory postsynaptic current (EPSC), paired‐pulse facilitation (PPF), and LTP with retention exceeding 10 s.

Ferroelectric polarization can also be integrated with volatile conductance‐tuning mechanisms to introduce long‐term plasticity into organic transistors. Wang et al. developed a ferroelectric/electrochemical modulated artificial synapse by incorporating an additional P(VDF‐TrFE) ferroelectric layer into the gate dielectric [78]. As presented in Figure 2e, at low operating voltages, anions within the gate dielectric electrostatically doped the channel, producing short‐term plasticity. Repeated low‐amplitude V_G_ pulses induced electrochemical long‐term plasticity with retention exceeding 100 s. When the gate potential surpassed the coercive field of the ferroelectric layer, non‐volatile ferroelectric states were established, which could persist for several hours.

In addition to ferroelectricity, the slow polarization dynamics of dipoles in dielectric layers have also been exploited to construct synaptic OFETs. Wang et al. reported a flexible synaptic OFET incorporating a DNTT thin film as semiconductor, a flexible organosilicone (Dow Corning 1‐2577) as dielectric, and a highly elastic polymer PDMS as support (Figure 2f) [79]. The slow kinetics of electrostatic dipoles formed by strongly polar hydroxyl (–OH) groups in the organosilicone dielectric produced pronounced hysteresis in the transfer characteristics. Leveraging this effect, the device successfully demonstrated STP as well as transitions from short‐term to long‐term synaptic behavior.

#### 4.1.3 Electrochemical Doping Switching

**FIGURE 3:**
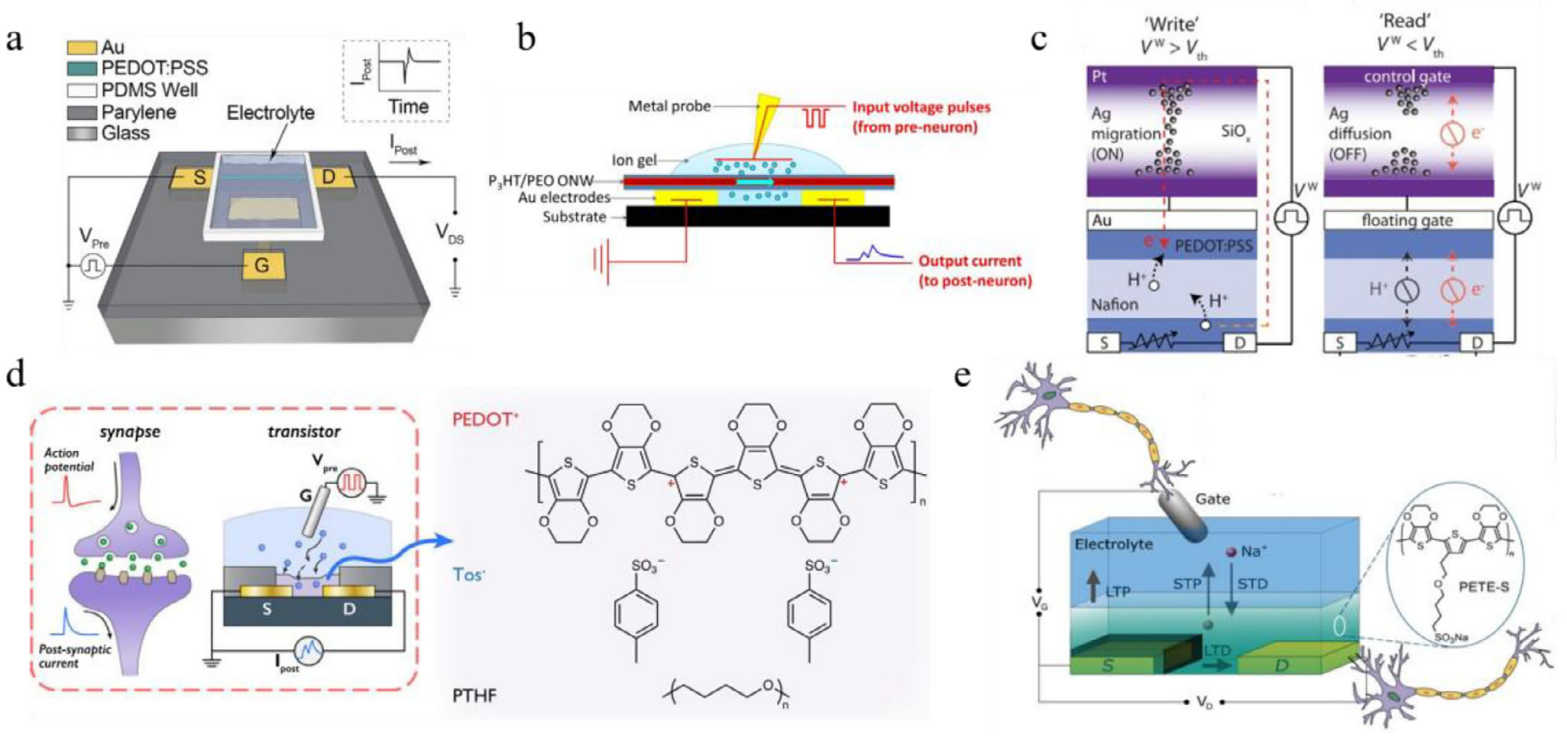
a) Schematic of an OECT artificial synapse exhibiting STP and its postsynaptic current response. Reproduced with permission [36]. Copyright 2015, WILEY‐VCH. b) An organic nanowire artificial synapse and its electrical wiring configuration. Reproduced with permission [80]. Copyright 2016, AAAS. c) CBM‐connected ionic floating‐gate (IFG) under read and write operations. The ON/OFF states of the CBM are controlled by increasing or decreasing the programming voltage V^W^. Adapted with permission [38]. Copyright 2019, AAAS. d) Analogy between a biological synapse and an OECT, along with the chemical structures of the channel materials PEDOT^+^, Tos^−^, and PTHF. Adapted with permission [87]. Copyright 2021, Springer Nature. e) Evolvable OECT artificial synapse, showing ion doping/de‐doping for STP and electropolymerization/overoxidation for LTP. Adapted with permission [88]. Copyright 2019, WILEY‐VCH.

OECT is a representative class of organic 3T artificial synapses operating via electrochemical doping. In such devices, electrochemical doping is achieved by modulating the gate potential to inject or extract ions into or from the channel material (Figure 1f). This process alters the redox state of the channel, thereby increasing or decreasing its conductivity through corresponding changes in the charge‐carrier density [8]. As illustrated in Figure 3a, Gkoupidenis et al. first demonstrated STP and related synaptic functions—including short‐term depression, adaptation, and dynamic filtering—in an OECT employing [poly(3,4‐ethylenedioxythiophene) doped with poly(styrene sulfonate] (PEDOT:PSS) as the channel and a NaCl aqueous solution as the electrolyte [36]. In this device, STP arises from the slow kinetics of the cations flowing between the PEDOT:PSS channel and the electrolyte. This mechanism has since been extended to a variety of organic channel materials and electrolytes, enabling the emulation of a broader range of short‐term synaptic behaviors [39, 80, 81, 82]. Notably, Xu and colleagues developed organic nanowire (ONW) artificial synapses composed of a poly(3‐hexylthiophene‐2,5‐diyl) (P3HT) inner core wrapped by a polyethylene oxide (PEO) sheath (Figure 3b) [80]. In this architecture, rapid ion diffusion between the ion gel and the PEO layer enabled STP, while restricted ion mobility within the P3HT core imparted LTP up to hundreds of seconds to the device.

Beyond transient plasticity, electrochemical systems can also achieve stable non‐volatile states. In 2017, van de Burgt et al. reported a novel electrochemical neuromorphic organic device (ENODe) which exhibited highly linear and reversible conductance modulation with retention times over 100 s during conductance‐state switching [37]. This device architecture, later termed an electrochemical random‐access memory (ECRAM) (Figure 1j), consisted of a PEDOT:PSS presynaptic electrode, a proton‐conducting Nafion electrolyte, and a PEDOT:PSS postsynaptic electrode partially reduced with poly(ethylenimine) (PEI). The ENODe operated in a battery‐like manner: application of a gate potential triggered complementary redox reactions at the pre‐ and postsynaptic electrodes, maintaining charge neutrality while enabling analog conductance tuning. Crucially, once programming was complete, the device remained disconnected from external charge sources, thereby suppressing charge compensation and allowing long‐term retention of the written conductance state up to 25 h.

Subsequently, Fuller et al. integrated a volatile conductive‐bridge memory (CBM) into the gate terminal of a redox‐gated polymer transistor, employing the same PEDOT:PSS electrodes and Nafion electrolyte as used in the ENODe [38]. As illustrated in Figure 3c, the CBM functioned as a selector. When the applied gate voltage V^W^ exceeded the CBM ON threshold V_th_, electrons were injected into the gate PEDOT:PSS, thereby modulating the conductance of the channel PEDOT:PSS. After programming, once V^W^ dropped below V_th_, the CBM switched to the OFF state, effectively blocking gate current. With the electronic pathway between the gate and channel interrupted, the channel conductance could be retained for up to 10 min. This architecture allowed the redox‐gated electrochemical transistor to be integrated into circuit arrays, enabling neuromorphic computing functions such as matrix multiplication and parallel weight updates.

The ECRAM architecture has since been further advanced through the incorporation of high‐performance or convenient organic channels, such as poly(2‐(3,3‐bis(2‐(2‐(2‐methoxyethoxy)ethoxy)ethoxy)‐[2,2‐bithiophen]‐5‐yl)thieno[3,2‐*b*]thiophene) p(g2T‐TT) and NDI‐bithiozale donor–acceptor copolymer (P‐3O), along with ion gel electrolyte films composed of ionic liquid (EMIM:TFSI) or 1‐ethylimidazolium bis(trifluoromethylsulfonyl)imide (EIM:TFSI) and (P(VDF‐HFP)) polymer matrix, achieving enhanced performance metrics including expanded dynamic range, accelerated switching speed, prolonged retention times, and improved thermal stability [83, 84].

#### 4.1.4 Other Switching Mechanism

Despite the significant progress achieved with the ECRAM device architecture in organic artificial synapses, alternative LTP mechanisms are also being actively explored. Gkoupidenis et al. demonstrated long‐term synaptic plasticity functions in an OECT employing poly(tetrahydrofuran) (PTHF)‐based PEDOT derivative (PEDOT:PTHF) as the channel material [85]. In this device, the steady‐state conductance could be retained for several hours, with the proposed non‐volatile tuning mechanism attributed to a structural collapse of the PEDOT composite that required a certain overpotential (e.g., 0.8 V) for re‐oxidation [86]. Subsequent work scaled down the channel geometry to accelerate the response time while preserving a retention time exceeding 200 min (Figure 3d) [87]. Further studies revealed that the non‐crystalline nature of PTHF promoted cation trapping within the channel, thereby imparting non‐volatility to the conductance states.

Another promising electrochemical approach for achieving LTP involves in situ electro‐polymerization of monomers within the channel. Gerasimov et al. developed an evolvable electrochemical transistor in which LTP arose from in situ electro‐polymerization and electrochemical overoxidation of additional semiconductor material sodium 4‐(2‐(2,5‐bis(2,3‐dihydrothieno[3,4‐b][1,4]dioxin‐5‐yl)thiophen‐3‐yl)ethoxy)butane‐1‐sulfonate (ETE‐S) within the channel, while the short‐term plasticity was achieved via electrochemical doping of the channel material PETE‐S [88]. As illustrated in Figure 3e, the device architecture integrated distinct mechanisms for short‐term and long‐term operation, with the molecular structure of the conducting channel enabling electrochemical modulation. In a subsequent study, the same group replaced ETE‐S with a structurally similar monomer (2‐(2,5‐bis(2,3‐dihydrothieno[3,4‐b][1,4]dioxin‐5‐yl)thiophen‐3‐yl) ethyl(2‐(trimethylammonio)ethyl) phosphate (ETE‐PC), which improved interaction with substrate and yielded long‐term plasticity with retention times exceeding 1000 s [89].

#### 4.1.5 Quantitative Benchmarking of Organic Synaptic Transistors

As discussed in the preceding subsections, a wide range of physical mechanisms—including charge trapping, dipole reorientation, electrochemical doping, and electropolymerization—has been exploited to realize synaptic behaviors in organic transistors. Given this diversity, a quantitative comparison of device‐level performance is essential for evaluating the technological maturity of each mechanism and for identifying their respective strengths and limitations. To contextualize the operating capabilities of organic synaptic transistors, Table 1 compiles representative long‐term synaptic performance metrics—including programming voltage, energy per event, programming pulse width, retention time, and dynamic range of demonstrated non‐volatile states—across major categories of organic devices. For reference, commonly reported metrics for 2D‐material–based synaptic transistors and CMOS analog synapses are also included. This benchmarking highlights the distinct operating regimes of organic neuromorphic devices and helps reveal performance gaps and opportunities that can guide future advances in materials development, device architecture, and system‐level integration. A more detailed discussion of these technological challenges is provided in the final section of this review.

**TABLE 1:** Representative performance metrics of organic transistor‐based synaptic devices compared with 2D‐material neuromorphic transistors and CMOS‐based synaptic implementations.

Device Type	Switching Mechanism	Programming Voltage	Energy per Event^*^	Programming Pulse Width	Retention Time	Dynamic Range	Ref.
OFET Synapses	Charge Trapping	1–50V	fJ∼µJ	10ms–0.5s	10–10^3^s	1.1–2.5	[67, 71, 90, 91]
	Ferroelectricity	3–30V	fJ∼nJ	100ms–0.5s	10–10^4^s	4–100	[76, 92, 93, 94]
OECT Synapses	Charge Trapping	0.3–2.5V	fJ	200ns–0.1s	10^2^–10^4^s	8–66	[74, 80, 87, 95, 96]
	Ferroelectricity	20–40V	pJ	100ms	10^4^s	10^4^	[78]
	Electro‐polymerization	0.5–2V	pJ	1s	10^2^–10^4^s	1.1–2.5	[88, 89, 97]
Organic ECRAM	Electrochemical Doping	0.01–1V	fJ–pJ	20ns–1s	10^2^–10^4^s	1.2–8	[37, 38, 83, 98, 99]
2D‐material Synapses	Various Mechanisms	0.5–50V	fJ–nJ	4.8ns–1s	10^2^–10^7^s	1.2–100	[100, 101, 102, 103, 104, 105, 106, 107, 108]
CMOS Synapse	—	0.5–5V	fJ–pJ	500µs/ ‐ (STDP)	10^4^–10^7^s	2–10	[13, 109, 110, 111]

* Including both reading and writing operations.

### 4.2 Stimulus‐Modulated Synaptic Plasticity in Organic Transistors

Beyond conventional electrical programming, neuromorphic behaviors in organic transistors can also be elicited by a wide range of external stimuli, including optical, mechanical, chemical and biochemical inputs, as well as their combinations. These alternative stimulus modalities expand the functional versatility of organic artificial synapses, enabling them to process multimodal information that can be integrated into intelligent systems to more closely emulate the biological sensory functions or served as bio‐interfaces and support advanced neuromorphic sensing applications. In this section, we review representative examples in which synaptic plasticity—both short‐term and long‐term—is modulated by different classes of stimuli at the device level, emphasizing how each modality interacts with the underlying device physics.

#### 4.2.1 Optical Stimuli

**FIGURE 4:**
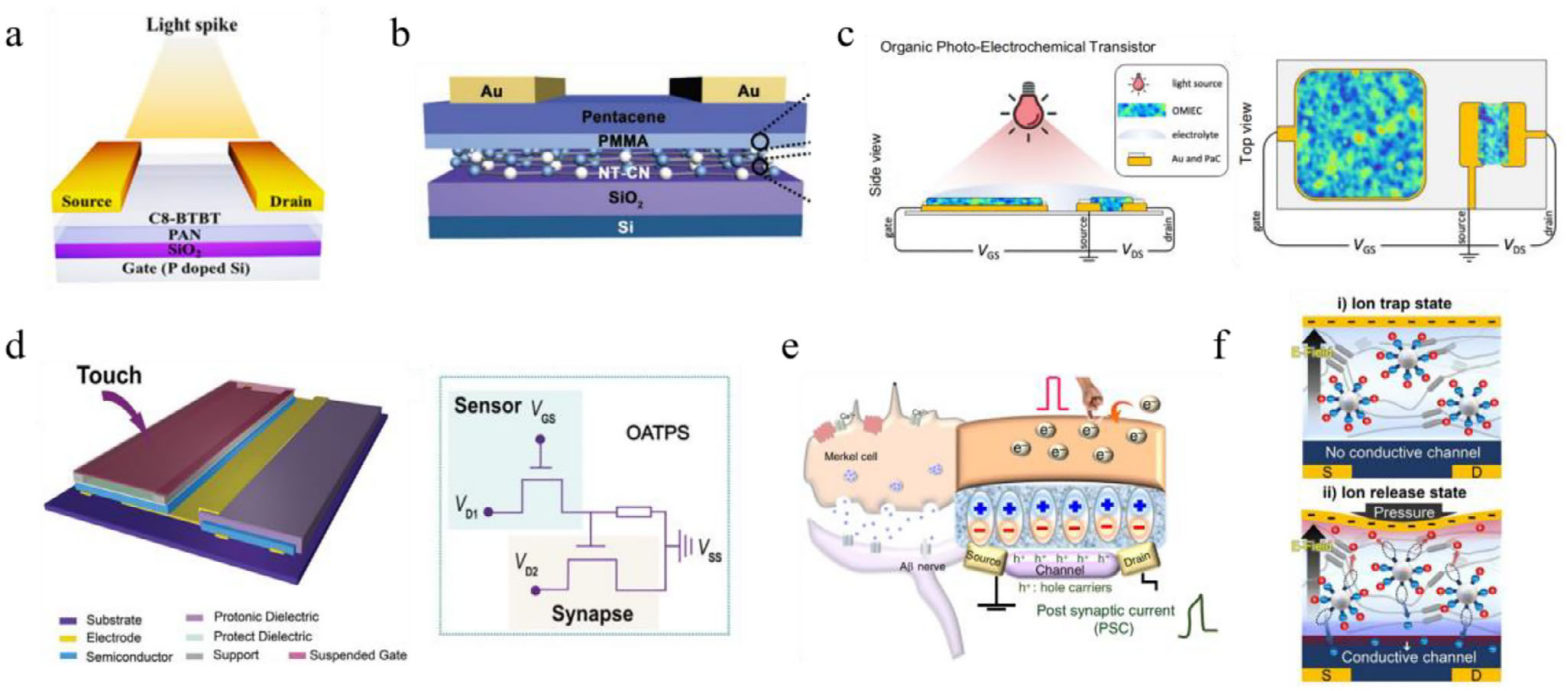
a) Simplified diagram of a C8‐BTBT OFET under light stimulation. Reproduced with permission [112]. Copyright 2018, American Chemical Society. b) Structure of the C_3_N_4_ photonic synapse device structure and its constituent materials. Adapted with permission [113]. Copyright 2020, WILEY‐VCH. c) Structure of the n‐OPECT, with p(C_6_NDI‐T) film coated on both the channel and gate electrode, immersed in phosphate‐buffered saline (PBS). Adapted with permission [116]. Copyright 2023, Springer Nature. d) Schematic illustration and equivalent circuit of the DOT‐TPE device showing pressure‐induced synaptic functions. Adapted with permission [120]. Copyright 2017, WILEY‐VCH. e) AiS‐TSO device structure mimicking a Merkel cell and its mechanical sensing mechanism. Reproduced with permission [124]. Copyright 2020, Springer Nature. f) Ion trapping and release states in NeuroMAT with and without tactile stimulation. Reproduced with permission [126]. Copyright 2023, AAAS.

Charge trapping has been effectively employed to construct organic optoelectronic artificial synapses. Huang and co‐workers reported an organic light‐stimulated synaptic transistor which exploited charge trapping at the organic semiconductor/dielectric interface of an OFET [112]. In their design, 2,7‐dioctyl[1]benzothieno[3,2‐b][1]benzothiophene (C8‐BTBT) was adopted as the active channel owing to its excellent semiconducting properties, while a polar polymer polyacrylonitrile (PAN) film served as the dielectric layer (Figure 4a). The strong polar groups in PAN introduced pronounced charge trapping effect at the OSC/PAN interface. Upon UV illumination of the C8‐BTBT channel, electron–hole pairs were generated, and light‐induced carriers gradually filled these interfacial traps. When the light stimulus was removed, the photo‐introduced charges were progressively retrapped, resulting in the restoration of channel conductance. Through this mechanism, the device successfully emulated synaptic behaviors, including both short‐term and long‐term plasticity, in response to optical inputs.

Floating‐gate architectures have also been leveraged in organic photonic artificial synapses to exploit charge‐trapping effects. Park et al. demonstrated a UV‐responsive organic floating‐gate synaptic transistor capable of selective detection and preprocessing of UV stimuli (Figure 4b) [113]. The device, termed carbon nitride‐based UV‐responsive synaptic transistor (CNUVS), integrated a UV‐responsive carbon nitride (C_3_N_4_) floating‐gate layer with SiO_2_ and PMMA gate insulators and a pentacene channel. Under UV irradiation, electron–hole pairs were generated in both the floating gate and the channel. Holes generated in C_3_N_4_ tunneled through the PMMA dielectric and were injected into pentacene, while electrons remained trapped within the floating gate. Once UV exposure ceased, excitons in the pentacene recombined rapidly, whereas the trapped electrons in the C_3_N_4_ film persisted for longer timescales. This disparity in recombination dynamics gave rise to STP behaviors in response to UV stimuli.

Optical modulation has also been incorporated into OECTs by exploiting the light sensitivity of the channel or by integrating light‐responsive materials into the gate electrode, enabling synaptic functionalities [114]. Mei et al. reported an OECT‐based optoelectronic artificial synapse in which electrochemical doping was modulated by light irradiation of the channel [115]. The device incorporated a photoactive poly(3‐hexylthiophene‐2,5‐diyl):[6,6]‐phenyl‐C61‐butyric acid methyl ester) (P3HT:PCBM) donor‐acceptor bulk‐heterojunction (BHJ) film within the channel and a lithium ion gel as electrolyte. Upon light absorption, the BHJ generated charge carriers that induced ion transport from the electrolyte into the channel for charge compensation. Anions surrounding the doped P3HT suppressed immediate charge recombination, thereby slowing current decay. This architecture enabled the device to respond sensitively to both light intensity and wavelength, while exhibiting photonic programming and electrical erasing of channel conductance. As a result, the OECT demonstrated both STP and LTP, with non‐volatile retention exceeding two hours.

In efforts to engineer regulatory gate electrodes, Inal and colleagues recently demonstrated synaptic functions in a single n‐type polymer‐gated photo‐electrochemical transistor (n‐OPECT) [116]. The device employed a thin film of a naphthalene‐1,4,5,8‐tetracarboxylic‐diimide–thiophene backbone polymer functionalized with triethylene glycol side chains anchored to the NDI unit via a six‐carbon spacer (p(C_6_NDI‐T)), which served as both the gate and channel material (Figure 4c). Upon light illumination, the polymeric electrodes exhibited reversible shifts in electrochemical potential and conductivity without involving faradaic reactions. When configured as an n‐OPECT, illumination enhanced the capacitance of the polymeric gate electrode, producing an additional potential drop across the channel–electrolyte interface and thereby driving the channel into a more doped state. This potentiometric photo‐gating effect enabled the n‐OPECT to emulate synaptic functions, including STP and LTP, with kinetics governed by the disparity between fast optical excitation and slower relaxation dynamics.

#### 4.2.2 Mechanical Stimuli

As one of the five traditionally recognized senses [117], tactile perception is of vital importance in the development of artificial sensory systems. Leveraging their inherent flexibility and stretchability, organic electronics have achieved significant progress in the construction of artificial skins [118, 119], and are now being advanced toward neuromorphic tactile sensing.

Synaptic tactile sensory functions can be realized through the integration of tactile sensors with synaptic devices [120, 121, 122, 123]. For instance, Zang and colleagues presented a dual‐organic‐transistor‐based tactile‐perception element (DOT‐TPE) which exhibited synaptic processing ability in response to pressure‐triggered electrical signals [120]. As shown in Figure 4d, the DOT‐TPE consisted of two OFETs: one for pressure‐sensing and the other one for synaptic signal‐processing. The sensing OFET employed a suspended‐gate structure, whereas the synaptic OFET adopted a conventional three‐terminal configuration with a proton‐conducting dielectric layer and PDPP3T as the semiconducting channel. The slow proton drift in the dielectric endowed the synaptic OFET with STP. During tactile perception, external pressure deformed the suspended gate of the sensing OFET, thereby modulating the capacitance of its dielectric and altering its channel conductivity. This change generated a sequence of voltage pulses at the gate of the synaptic OFET, which, in turn, enabled neuromorphic tactile sensing through the cooperative action of the two transistors.

In a more recent study, Lee et al. presented a flexible, artificial, intrinsic‐synaptic tactile sensory organ (AiS‐TSO) which demonstrated synaptic responses to pressure stimuli such as finger touch in a monolithic device [124]. The AiS‐TSO was based on a ferroelectric organic field‐effect transistor comprising a dielectric layer of barium titanate nanoparticle/P(VDF‐TrFE) composite, a pentacene semiconducting channel, and a polyimide (PI) substrate. The device was gated through a triboelectric–capacitive coupling effect, as illustrated in Figure 4e. Mechanical contact between skin and the receptive gate electrode induced negative charge accumulation at the gate due to the difference in electron affinity between the skin and the receptive material. These pumped triboelectric charges generated a tribo‐capacitive potential, which strengthened dipole alignment within the ferroelectric dielectric and thereby increased channel conductance. Upon removal of the tactile stimulus, the negative charges on the triboelectric layer initially disrupted charge equilibrium, transiently enhancing dipole alignment and producing an increased postsynaptic current. This current gradually decayed as the tribo‐capacitance was discharged. while the remnant polarization of the ferroelectric nanoparticles continued to modulate channel conductance, enabling the AiS‐TSO to exhibit long‐term synaptic behaviors under tactile inputs.

Triboelectric effects have also been harnessed in OECT‐based tactile synapses. For instance, Lee et al. demonstrated a triboelectric‐driven synaptic OECT by monolithically integrating an OECT synapse with an ion gel‐based triboelectric nanogenerator (TENG) [125]. This device employed a PEDOT:PSS channel, while the ion gel—composed of the ionic liquid EMIM:TFSI and a P(VDF‐HFP) polymer matrix—served simultaneously as the gate dielectric and triboelectric layer for tactile reception. Upon tactile stimulation of the receptor, triboelectric charges were generated at the ion gel interface due to electrostatic interactions with the contacting material. This triboelectric potential drove electrochemical doping in the PEDOT:PSS channel, thereby modulating its conductance. Following removal of the tactile input, the device gradually returned to electrochemical equilibrium through ion redistribution, restoring the channel conductance to its initial state. This process, analogous to STP in conventional OECTs, allowed the device to reproduce synaptic responses specifically to tactile stimuli.

Novel neuromorphic tactile sensory mechanisms have also been explored in recent studies. Kim and colleagues developed a neuro‐inspired, monolithic artificial tactile neuron (NeuroMAT) which exhibited nonintrusive and augmented memory based on ion trap and release dynamics (iTRD) [126]. The device employed an iTRD ion gel composed of silica microparticles coordinated with the ionic liquid EMIM:TFSI and dispersed in a thermoplastic polyurethane (TPU) matrix as the electrolyte, together with polymer semiconductor poly[2,5‐bis(2‐decyltetradecyl)‐3‐5‐(thieno[3,2‐b]thiophen‐2‐yl)thiophen‐2‐yl‐6‐(thiophen‐2‐yl)pyrrolo[3,4‐c]pyrrole‐1,4(2H,5H)‐dione] (PDPPTT) as the channel. In the absence of pressure, the transistor exhibited negligible response to the gate voltage pulses due to the presence of ion‐trap states. Under combined mechanical stimulation and applied gate bias, however, pressure‐induced release of trapped ions enabled volumetric electrochemical doping of the semiconductor, resulting in pressure‐sensitive current modulation (Figure 4f). This mechanism allowed the NeuroMAT to emulate both STP and LTP exclusively under tactile stimulation in a monolithic device, with retention times exceeding 3600 s.

#### 4.2.3 Chemical and Biochemical Stimuli

Organic electronics, particularly organic thin‐film transistors, have already achieved significant success in chemical and biosensing applications over the past decades [127, 128, 129, 130]. Building on these advances, neuromorphic chemical and biochemical sensing based on organic electronics has emerged as a promising direction for emulating olfactory and gustatory systems, enabling seamless bio‐interfaced signal processing and expanding the scope of artificial sensory platforms.

**FIGURE 5:**
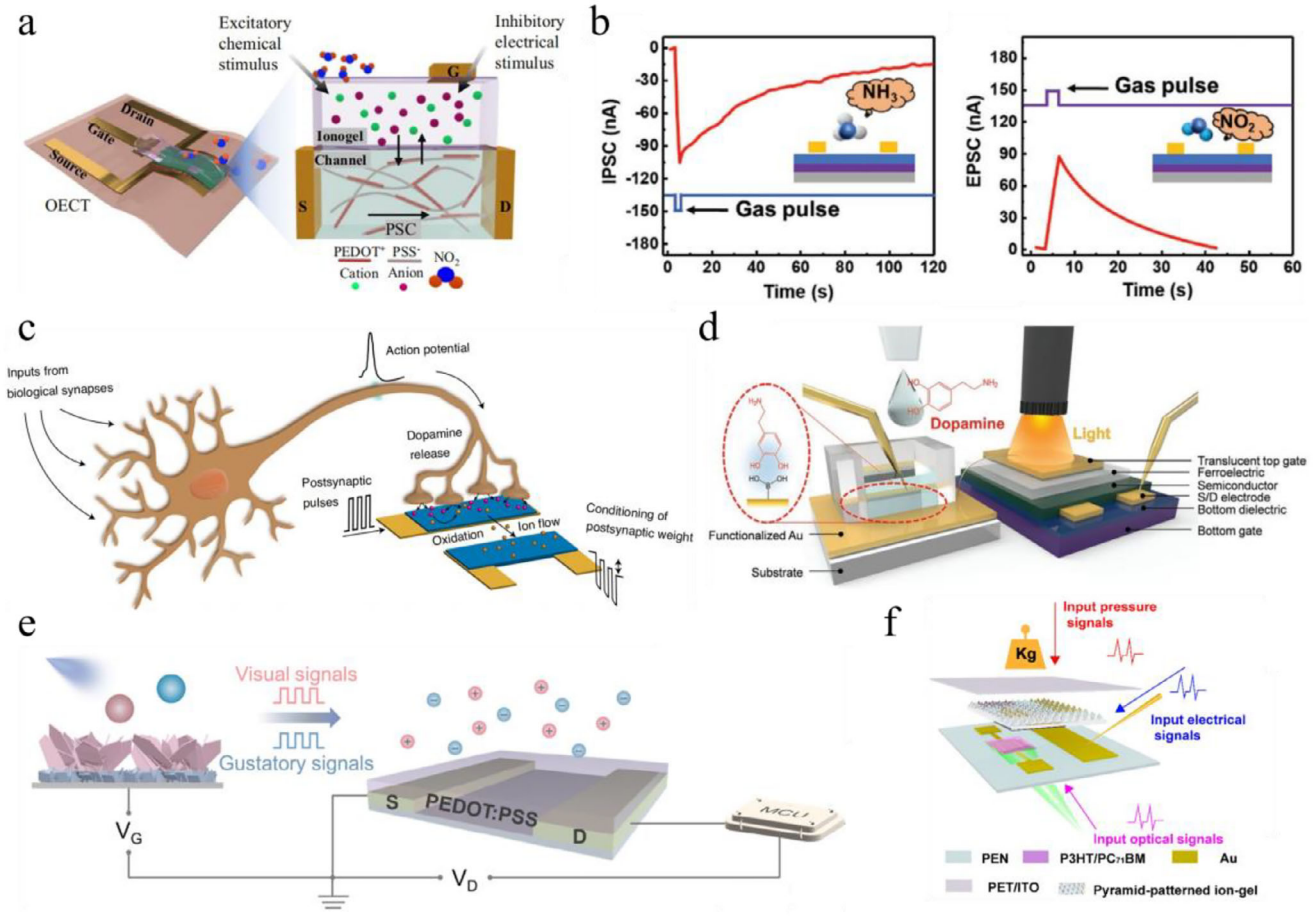
a) Schematic illustration of the NO_2_ gas‐ and electrically modulated synaptic OECT device and its doping/de‐doping mechanism. Reproduced with permission [131]. Copyright 2023, Springer Nature. b) Inhibitory and excitatory postsynaptic responses of the device to NH_3_ and NO_2_ signal, respectively. Adapted with permission [132]. Copyright 2024, Wiley‐VCH. c) Schematic of the neurotransmitter‐mediated neuromorphic device and its postsynaptic responses during DA release. Reproduced with permission [133]. Copyright 2020, Springer Nature. d) Photonic artificial synapse with a DA‐responsive extended gate. Reproduced with permission [146]. Copyright 2021, Wiley‐VCH. e) Schematic of the OPECT‐based artificial multisensory integration system. Adapted with permission [147]. Copyright 2025, Wiley‐VCH. f) MASS device and synaptic structure for the fusion of optical, pressure, and electrical signals. Reproduced with permission [148]. Copyright 2023, American Chemical Society.

Neuromorphic gas sensing, in particular, has been explored as a route toward artificial olfactory systems. For instance, Chouhdry and colleagues proposed an organic artificial chemosensory neuronal synapse (ACNS) capitalizing on a flexible OECT incorporating a chemoreceptive ion gel [131]. As illustrated in Figure 5a, this artificial synapse consisted of a PEDOT:PSS active channel, an ion gel composed of the ionic liquid EMIM:TFSI, poly(ethylene glycol) diacrylate (PEGDA) monomer, and a 1‐hydroxycyclohexyl phenyl ketone photoinitiator, serving simultaneously as the chemical receptive medium and the gate electrolyte. Nitrogen dioxide (NO_2_) gas, upon dissolving in the ionic liquid, interacted with the cations in the ion gel, leading to anions migration into the channel via the negative gating effect in the ion gel. As a result, this process increased the conductance of PEDOT:PSS under chemical pulse stimulation, enabling the emulation of chemoreceptive synaptic functions. Due to the stable bonds between NO_2_ and cations in the ion gel, synaptic weight was maintained for a few hundred seconds after the cessation of gas pulses, demonstrating long‐term plasticity. The application of an inhibitory electrical stimulus facilitated the expulsion of anions from the channel, allowing reversible modulation of synaptic weight.

In another example, excitatory and inhibitory synaptic behaviors were realized in a chemically sensitive transistor through exposure to different gas pulses. Wu et al. reported an olfactory synaptic OFET in which long‐term potentiation and depression were achieved through oxidative and reductive gas stimulations, respectively [132]. This artificial synapse employed a bottom‐gate top‐contact architecture with the p‐type semiconductor poly‐diketo‐pyrrolopyrrole‐selenophene (PTDPPSe‐5Si) as the active channel and a Cytop‐decorated SiO_2_ dielectric layer. The bulky siloxane side chains and the nanowire‐like surface morphology of the spin‐coated semiconducting film facilitated efficient gas absorption and surface charge transfer. As illustrated in Figure 5b, upon exposure to reducing gas NH_3_, the source‐drain current rapidly decreased due to the strong electron‐donating ability of NH_3_, which introduced hole traps and reduced the density of effective carriers in the channel. Conversely, exposure to the oxidizing gas NO_2_, with its strong electron‐accepting properties, generated electron traps that increased the effective carrier density in the channel. These complementary responses enabled the device to emulate both STP and LTP, as well as excitatory‐inhibitory balance, under gas pulse inputs.

Biosensing has been extensively investigated in organic transistors, and their intrinsic biocompatibility, including responsiveness to biomolecules and stable operation in aqueous biological environments, provides unique advantages for developing biochemical‐mediated neuromorphic devices. In 2020, Keene et al. pioneered an organic biohybrid synapse mediated by dopamine (DA) signals [133]. In this work, a dopaminergic presynaptic domain of PC‐12 cells was interfaced with a redox‐gated OECT employing PEDOT:PSS as the active material (Figure 5c). DA exocytosed by PC‐12 cells was oxidized at the PEDOT:PSS postsynaptic gate electrode under applied voltage pulses, altering its charge state and consequently modulating the conductance of the PEDOT:PSS channel. Washing away the accumulated DA at the gate restored the channel conductance to its initial state. Through this mechanism, LTP was achieved exclusively in the presence of DA, demonstrating a neurotransmitter‐mediated neuromorphic device. This biochemical‐mediated concept, in which LTP can be selectively induced upon the recognition of specific biomolecular signals, has further been extended to the construction of artificial synapses responsive to glucose [134], serotonin (5‐HT) [135, 136], glutamate [137], and L‐cysteine [138].

#### 4.2.4 Multimodal Stimuli

In biological neural networks, multisensory integration (MSI) synthesizes information from different sensory modalities to generate more accurate perceptual representations of events and to facilitate optimal decision‐making [139]. Similarly, in artificial systems, the ability to simultaneously respond to multiple types of stimuli associated with the same event is highly advantageous for advancing intelligent technologies such as robotics, wearable electronics, and human–machine interfaces [140, 141, 142]. By enabling sensing and processing of diverse inputs directly at the device level, multimodal fusion can improve classification accuracy and recognition performance while reducing the need for additional external chips or complex circuit integration [143, 144, 145]. Owing to their inherent material versatility and compatibility with diverse sensing modalities, organic neuromorphic devices provide an especially promising platform for implementing such multimodal integration.

One practical approach to achieving multimodal sensing is the integration of extended‐gate structures. For instance, Lee and co‐workers reported an OFET‐based dual‐gate organic synaptic transistor (DGOST) platform capable of simultaneously detecting dopamine and light (Figure 5d) [146]. This device consisted of a photoconductive organic semiconductor poly[[4,8‐bis[5‐(2‐ethylhexyl)‐2‐thienyl]‐benzo[1,2‐b:4,5‐b′]dithiophene‐2,6‐diyl][2‐(2‐ethyl‐1‐oxohexyl)‐thieno[3,4‐b]thiophenediyl]] (PBDTTT‐C‐T), sandwiched between a bottom SiO_2_/Si gate and a top P(VDF‐TrFE)/Au gate structure. In addition, a dopamine‐responsive extended‐gate electrode (DREGE) functionalized with self‐assembled monolayers (SAMs) of boronic acid was connected to the top‐gate electrode via silver paste and gold wire. The DGOST‐DREGE demonstrated synaptic responses to both dopaminergic and optical stimuli. For dopaminergic inputs, successive additions of 1×10^−3^ M DA solution into the PBS container led to enhanced sensitivity and rate of change in current, owing to charge density modulation at the extended‐gate electrode through esterification‐based specific binding. For optical inputs, repetitive exposure to polychromatic light on the translucent top gate significantly enhanced the postsynaptic current through exciton generation and charge separation in the channel, producing long‐term potentiation under repeated light pulses. The ferroelectric polarization of the P(VDF‐TrFE) top‐gate dielectric also played a critical role in the synaptic responses. Notably, synergistic multimodal effects were observed, where light exposure amplified the postsynaptic current response induced by the same DA injection, mimicking memory consolidating effect.

The integration of multimodal sensing and processing within a single monolithic device can significantly improve fabrication compatibility, integration density, and conductance matching [74], offering greater efficiency and convenience compared to extended‐gate approaches. For example, Huang et al. developed a multimodal OPECT which emulated the visual‐gustatory MSI in a single device (Figure 5e) [147]. The OPECT consisted of a BiVO_4_/WO_3_ heterojunction photogate and a PEDOT:PSS polymeric channel. The memory behavior originated from two mechanisms: time delays in photoexcited electron–hole recombination due to the band barrier at the heterojunction interface, and the slow ion migration/relaxation dynamics associated with volumetric doping of the polymeric channel. The device exhibited independent responses to optical and chemical stimuli, demonstrating both optical synaptic behaviors and pH sensitivity. Moreover, synergistic multimodal STP was achieved by combining optical and chemical inputs: responses to fixed‐intensity light decreased at acidic pH (down to 3.0) and increased at alkaline pH (up to 11.0), mimicking gustation‐dependent inhibition or enhancement of visual perception. In addition, typical MSI characteristics were successfully mimicked, including “super‐additive response,” “inverse effectiveness effect” and “temporal congruency.”

In another example, Shao and colleagues reported a highly integrated organic multimodal artificial sensory synapse (MASS) capable of emulating synaptic behaviors in response to optical, electrical and tactile stimuli (Figure 5f) [148]. The device incorporated a photosensitive channel composed of P3HT blended with [6,6]‐phenyl‐C71‐butyric acid methyl ester (PC_71_BM)], a pyramid‐patterned ion gel dielectric based on EMIM:TFSI and P(VDF‐HFP) for pressure sensitivity, an indium tin oxide/poly(ethylene‐2,6‐naphthalenedicarboxylate) films (ITO/PEN) top gate and a Cr/Au side gate. Distinct synaptic functions were demonstrated for each stimulus: optical STP and LTP arising from exciton generation, separation, and hole trapping at the P3HT/PC_71_BM interface; electrical STP and LTP due to p‐doping of the channel, electrolyte ion dynamics, and ion trapping at the ion gel/channel interface; and tactile STP and LTP induced by pressure spikes through mechanisms analogous to electrical inputs. Synergistic effects were also examined: combined electrical and optical input enhanced memory responses, while the synchronous input of electrical and tactile signals showed no significant influence. Furthermore, the device successfully mimicked higher‐order cognitive functions, including associative learning and dynamic memory modification, within a single architecture. This work highlights the potential of organic multimodal synapses as compact and versatile platforms for bioinspired multisensory processing.

## 5 Organic Artificial Neuron

In addition to organic artificial synapses, organic artificial spiking neurons, most notably those based on OECT circuits, have recently attracted growing research interest due to their resemblance to the chemically mediated signaling processes of biological systems [40, 44, 149, 150, 151]. These components, commonly referred to as organic electrochemical neurons (OECNs), are designed to reproduce the spiking behaviors of biological neurons, with a primary focus on signal integration for spike generation and a threshold‐dependent excitation to emulate the all‐or‐none principle. OECNs hold great promise as biohybrid interfaces for neuromorphic perception, owing to their intrinsic flexibility, biocompatibility, and multimodal sensing capabilities, including biochemical responsiveness. In the following, we briefly review recent developments in OECNs and discuss how their spiking dynamics arise from, and are influenced by, the physical and material parameters of the underlying OECTs.

### 5.1 Recent Advances in OECNs

**FIGURE 6:**
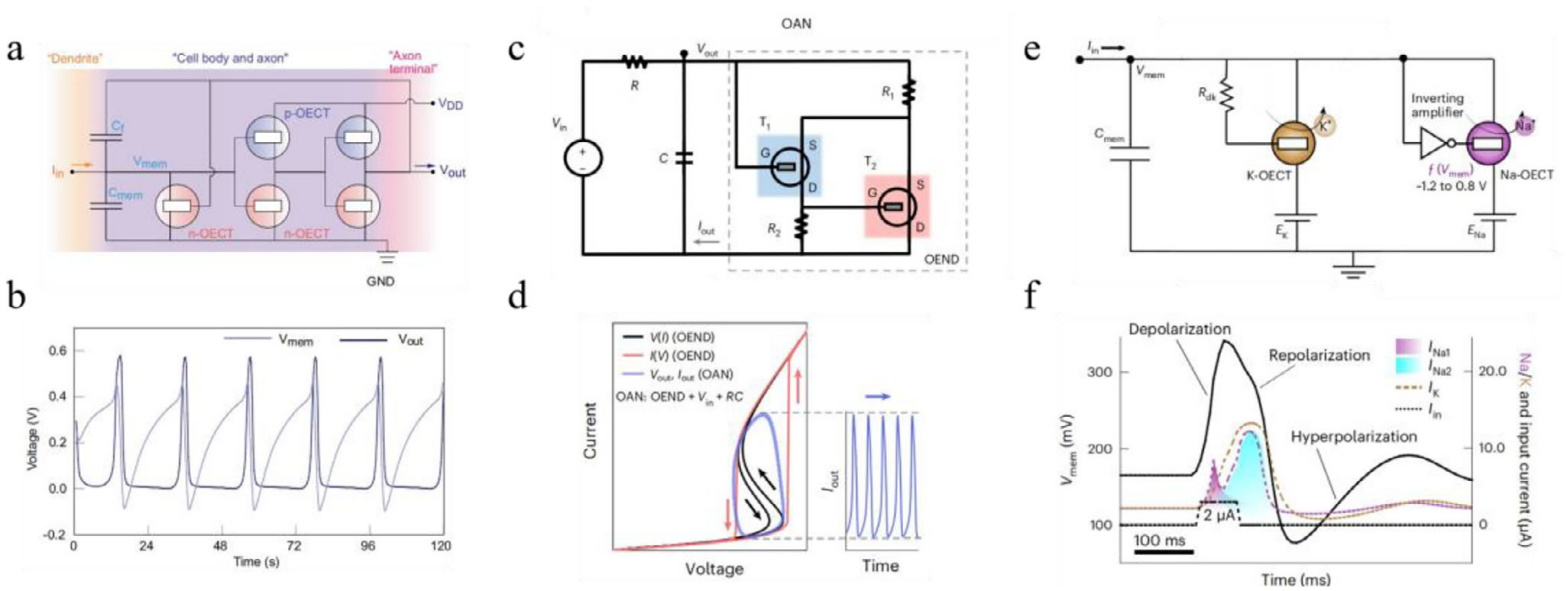
a) Schematic of an OECN based on an A‐H circuit. b) Spiking behaviors of the OECN. Reproduced with permission [89]. Copyright 2022, Springer Nature. c) Circuit diagram of the OEND‐based OECN. d) Nonlinear dynamics of the OEND and its oscillatory output. Adapted with permission [152]. Copyright 2022, Springer Nature. e) Circuit diagram of the c‐OECN comprising Na^+^‐ and K^+^‐based OECTs. f) AP waveforms generated by the c‐OECN. Adapted with permission [153]. Copyright 2023, Springer Nature.

The leaky integrate‐and‐fire (LIF) model is a simple yet widely adopted spiking neuron framework, designed to capture essential neuronal dynamics such as temporal integration, membrane leakage, and threshold‐triggered firing. In 2022, Harikesh et al. reported the first all‐printed LIF‐type organic electrochemical neuron which exhibited ion‐mediated spiking behaviors [89]. This OECN implemented an Axon‐Hillock (A‐H) neuromorphic circuit composed of complementary OECTs, utilizing P(g_4_2T‐T) and poly(benzimidazobenzophenanthroline) (BBL) as the p‐type and n‐type semiconductors, respectively (Figure 6a). As illustrated in Figure 6b, the device was capable of integrating current inputs and generating voltage spikes analogous to biological neurons, with spike frequency modulated by both the input current and the ion concentration in the electrolyte. The biohybrid integration of the OECN was further demonstrated using Venus Flytraps: high current inputs produced high‐frequency voltage spikes that induced lobe closure, whereas low current inputs generated low‐frequency spikes insufficient to trigger closure. In addition, the OECN was coupled with organic electrochemical synapses, enabling associative learning through spike‐timing‐dependent plasticity (STDP).

Implementations of the LIF neuron model often rely on many‐element oscillatory circuits, which are bulky and limited in their ability to reproduce complex neuronal dynamics. To overcome these limitations, Sarkar and colleagues developed an organic artificial neuron (OAN) built on a compact organic electrochemical non‐linear device (OEND) for local and in situ neuromorphic sensing and bio‐interfacing [152]. The OEND consisted of two p‐type OECTs: a depletion‐mode transistor based on a PEDOT:PSS channel and an enhancement‐mode transistor based on a p(g2T‐TT) channel (Figure 6c). Unlike solid‐state circuits, the PEDOT:PSS channel directly interfaced with the aqueous electrolyte, mimicking the extracellular environment of biological neurons. The OEND exhibited S‐shaped negative differential resistance (S‐NDR) characteristics, and when coupled to a simple resistor‐capacitor element, the resulting OAN produced oscillatory current spikes under constant voltage bias (Figure 6d). The OAN reproduced several biophysically relevant neuronal features, including excitability tuned by electrochemical oscillations, spike latency, temporal integration, and noise‐coupled excitability. The OAN also demonstrated concentration‐dependent modulation of firing frequency, responsiveness to biomolecular species such as dopamine, and ion‐specific oscillatory activity (e.g., Na^+^ or K^+^). Finally, biohybrid neurons were constructed by incorporating an epithelial cell membrane between the gate and PEDOT:PSS channel, showing real‐time modulation of neuronal excitability by biological membranes, highlighting the capability of the OAN for in situ bio‐interfacing.

More biorealistic neuron models are being realized using OECNs to emulate complex neuronal dynamics beyond the capabilities of simple integrate‐and‐fire circuits. Harikesh et al. reported a conductance‐based organic electrochemical neuron (c‐OECN) that exploits the ion‐tunable antiambipolar behavior of the ladder‐type polymer BBL [153]. When employed as the channel material in OECTs, BBL exhibits a stable and reversible Gaussian‐shaped transfer curve, similar to voltage‐controlled NDR. As shown in Figure 6e, the c‐OECN consisted of two BBL‐based OECTs, analogous to the Na and K ion channels and batteries in the Hodgkin‐Huxley model. This architecture enabled the generation of continuous AP‐like spiking under constant current input, reproducing essential features of biological neurons, including depolarization, repolarization, and hyperpolarization (Figure 6f). Importantly, the c‐OECN reproduced most known neural features (15 out of ∼20), exhibited tonic spiking at bioplausible frequencies up to ∼100 Hz, and demonstrated stochastic responses under noise. As a proof of concept for biointegration, the device was operated as an event‐based Na^+^ sensor to trigger vagus nerve stimulation in mice. This demonstration underscores the potential of c‐OECNs for future closed‐loop physiological regulation.

In addition to emulating a broader range of neuronal spiking behaviors, significant progress has also been made in enhancing the performance of OECNs. Recently, Yao et al. reported a LIF‐type OECN with a markedly expanded spiking frequency range and reduced device footprint, advancing its application in neuromorphic perception systems [149]. This OECN was realized using high‐performance vertical OECT complementary circuits, constructed from an innovative n‐type OMIEC Homo‐gDPPTz and a p‐type gDPP‐g2T polymer, which together enabled well‐balanced complementary inverter operation. Owing to the vertical device architecture and optimized channel materials, the OECN achieved a compact footprint (<37 mm^2^) and demonstrated a calibratable firing frequency spanning 0.130 to 147.1 Hz, over 50 times broader than that of conventional Axon‐Hillock OECT circuits (<2.4 Hz). Such a wide range makes it possible to mimic the diverse firing rates of biological neurons, from slow vasoconstrictor neurons (<1 Hz) to fast‐spiking cortical neurons (>100 Hz). The OECN was further integrated with pressure and strain sensors, as well as an organic artificial synapse, to construct a neuromorphic tactile perception system. Within this system, tactile signals were transduced into frequency‐dependent spikes and subsequently converted into postsynaptic responses, closely emulating the sensing–encoding–processing loop of biological neural perception.

### 5.2 Parameter‐Dependent Spiking Dynamics in OECNs

Although the circuit topologies of OECN architectures vary, their spiking dynamics are fundamentally governed by the electrochemical operation of the constituent OECTs and by the RC elements. As illustrated by Belleri et al. [154], the drain current of an OECT can be quantitatively described using an extended Bernads‐Malliaras model which incorporates energy disorder, channel‐length modulation, and ionic effects: [155, 156, 157, 158]

(1) $$I_{D}=\frac{\Gamma }{\gamma }V_{P}^{2-\gamma }\left[∥V_{S}-V_{G}+V_{TH}{∥}^{\gamma }-∥V_{D}-V_{G}+V_{TH}{∥}^{\gamma }\right]\left(1+\lambda V_{SD}\right)$$

where

(2) $$\Gamma =\frac{W}{L}t\mu C_{V}$$

(3) $$V_{P}=\frac{qp_{0}}{C_{V}}$$

(4) $$\gamma =\frac{E_{0}}{k_{B}T}+1$$

(5) $$V_{TH}=V_{P}-V_{SH}+V_{ISM}$$

Here, *W*, *L*, and *t* denote the channel width, length and thickness, respectively; *µ* is the hole mobility; and *C_V_* is the volumetric capacitance. *q* is the elementary charge, and *p_0_* represents the intrinsic doping of the semiconductor. The disorder parameter *E_0_* defines the energetic tail of the density of states, and, together with the Boltzmann constant *k_B_* and the temperature T, determines the parameter *γ*. The factor *λ* accounts for channel length modulation, while *V_TH_* is the threshold voltage. The terms *V_SH_* and *V_ISM_* capture, respectively, ion‐concentration–dependent shifts and ion‐selective membrane–induced potentials. *V_S_*, *V_D_*, *V_G_* correspond to the applied source, drain and gate voltages. The || · || operator is defined as:

∥x∥=xifx>0

∥x∥=0ifx<0

In OMIECs with low energetic disorder (*γ* ≈ 2), Equation 1 simplifies to:

(6) $$I_{D}=g_{m}\left[∥V_{S}-V_{G}+V_{TH}{∥}^{\gamma }-∥V_{D}-V_{G}+V_{TH}{∥}^{\gamma }\right]$$

where $g_{m}=\frac{\Gamma }{\gamma }V_{P}^{2-\gamma }$ is the normalized transconductance (∂*I_D_*/∂*V_SG_*)/(*V_S_* − *V_D_*). This device‐level model enables quantitative predictions of how material parameters (*µ*, *C_V_*, *p_0_*, *E_0_*), ionic environment (electrolyte concentration, interfering ions), and geometry (W/L/t) jointly shape neuronal behavior, including firing frequency, excitability, and ion regulation.

Belleri et al. applied this model to analyze an OAN based on an OEND, identifying how the OECT parameters controlled nonlinear switching (S–NDR) and thus the emergence of spiking [154]. For instance, reproducing S‐NDR required the threshold voltages of the two OECTs to satisfy *V_TH1_* >0 V, *V_TH2_* <0 V, and *V_TH1_* >|*V_TH2_* | (see Figure 6c). Numerical simulations further reveal that the spiking frequency *f_spike_* was tunable across orders of magnitude by engineering device parameters: *f_spike_* increased with decreasing *V_TH1_*, *g_m1_*, *C_V1_*, *R_2_* and with reductions in geometry *W_1_*, *W_2_*, *L_2_*, *t_1_*, and *t_2_*, while increasing *C_V2_* and *L_1_* elevated the frequency. A maximum *f_spike_* ≈150 Hz was predicted under optimized conditions. Similarly, neuron excitability grew with larger *g_m1_*, *g_m2_*, *C_V1_*, *C_V2_*, *R_1_* and *R_2_* and diminished with increasing *V_TH1_* and *V_TH2_*. Because the threshold voltage shifts with electrolyte composition (Equation 5), this model also captures how ionic environments modulate neuronal characteristics. The *V_ISM_* term further incorporates the influence of ion‐selective membranes, enabling selective responses to specific ions (e.g., K^+^, Na^+^, Ca^2+^). Using this framework, simulations successfully reproduced ion‐dependent firing thresholds, spiking frequency modulation across physiological concentrations, and ion selective spiking, illustrating how OANs might serve as neuromorphic ion sensors or biohybrid interfaces.

Overall, this analytical framework provides a powerful tool for guiding the design of organic artificial neurons. By linking physical quantities—such as mobility, volumetric capacitance, and device geometry—to emergent neuronal properties, it enables rational material and device optimization to engineer neurons with targeted frequency ranges, excitability profiles, ion sensitivity, and power consumption, thereby expanding the functional design space for organic neuromorphic electronics.

## 6 Neuromorphic Applications

Although much of the progress in organic neuromorphic electronics remain focused on demonstrating neuro‐mimetic behaviors and proof of concepts, recent advances have increasingly extended their scope toward functional neuromorphic applications. On one front, neuromorphic device have been employed as computing units for brain‐inspired paradigms such as neural network algorithms and reservoir computing. On another, they have been integrated into bio‐inspired sensory platforms, enabling in‐sensor computing, multisensory integration, and sensorimotor integration for applications including artificial skins and robotics. In particular, the intrinsic softness and biocompatibility of organic materials have facilitated the development of neuromorphic bio‐integrated interfaces capable of directly interacting with living tissues.

Together, these demonstrations underscore the distinctive ability of organic neuromorphic systems to seamlessly combine sensing, memory, and processing within a single architecture, achieving significantly lower energy consumption and simpler circuitry compared to conventional approaches. Importantly, such systems have exhibited both predefined responses and learned behaviors, demonstrating hardware‐based intelligence. In the following sections, we review representative neuromorphic applications across these three categories, outlining both the capabilities realized to date and the opportunities they create for future intelligent, flexible, and bio‐compatible systems.

### 6.1 Neuromorphic Computing Hardware

#### 6.1.1 Hardware‐Based Neural Network

**FIGURE 7:**
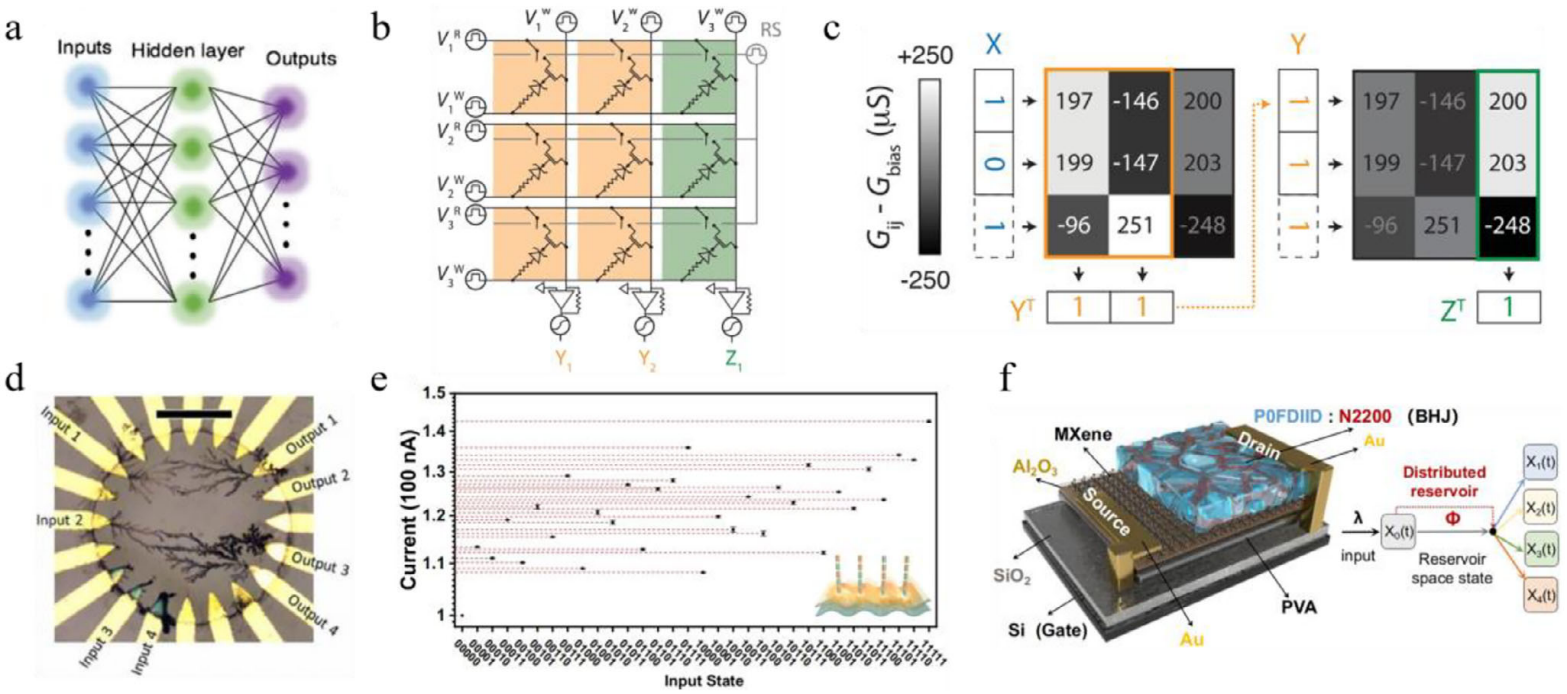
a) Schematic illustration of a two‐layer neural network with one hidden layer. Adapted with permission [164]. Copyright 2016, WILEY‐VCH. b) Voltage‐programmed crossbar array based on ECRAMs for in‐memory MVM operations. c) XOR classification based on a 3 × 3 ECRAM crossbar. Reproduced with permission [38]. Copyright 2019, AAAS. d) Optical microscope image of the dendritic fiber‐based reservoir with four input channels and four output channels. Reproduced with permission [175]. Copyright 2021, AAAS. e) Experimental read‐current responses generated by thirty‐two 5‐bit optical pulse trains ranging from (00000) to (11111). Reproduced with permission [179]. Copyright 2023, Springer Nature. f) Device structure of the VOFET‐DR and schematic representation of its distributed reservoir states. Reproduced with permission [181]. Copyright 2024, Springer Nature.

Traditional computing architectures are based on the von Neumann model, where computation and storage are physically separated. Continuous data transfer between memory and processing units creates a fundamental performance bottleneck, as energy and time are largely consumed during data movement. This limitation, commonly referred to as the “memory wall” [2, 3], not only constrains system scalability but also makes neural network algorithms particularly costly in terms of efficiency (Figure 7a). In‐memory computing offers a promising strategy to overcome this bottleneck by enabling certain computational tasks to be performed directly within memory. A representative example is the crossbar array, in which resistive memory devices located at each intersection can execute weight updates in parallel and perform matrix–vector multiplication (MVM) inherently in memory (Figure 7b) [22]. Leveraging Kirchhoff's and Ohm's laws, forward inference can be accomplished in a single computational step. Moreover, learning efficiency is enhanced since backpropagation can be accelerated by implementing rank‐one outer product updates in parallel within the same crossbar structure [159].

Artificial synapses with tunable non‐volatile conductance have been extensively investigated as promising building blocks for hardware‐based artificial neural networks (ANNs) [9, 160, 161, 162]. For analog forward‐inference neural network, linearity, symmetry, and precision in conductance updates, including low noise, high repeatability, wide dynamic range, and a large number of accessible states (>100) are directly linked to achieving AI task accuracies comparable to ideal numerical simulations [163, 164]. Meanwhile, properties such as state retention, switching energy, switching speed, endurance, scalability, CMOS compatibility, and environmental stability play a crucial role in determining the feasibility of real‐world hardware implementations [8]. Among the various material platforms, organic artificial synapse have garnered particular interest as they combine several advantageous features: low‐voltage operation and high tunability to meet system's demands, along with intrinsic flexibility, stretchability and biocompatibility that render them especially suitable for biohybrid applications [8, 40].

Organic synaptic transistors have been continually advanced as multi‐state non‐volatile memories for constructing crossbar arrays to accelerate ANN operations, showing steady improvements in device performance toward practical applications. For instance, van de Burgt et al. developed an ENODe which demonstrated more than 500 distinct non‐volatile states with a switching energy below 10pJ (for 10^3^ µm^2^ devices) [37]. The excellent linearity and low noise of ENODe further enabled circuit‐level simulations to achieve 97% classification accuracy on the MNIST dataset. Building on this, Fuller et al. demonstrated an IFG memory array based on CBM‐connected organic redox transistors (Figure 3c), achieving sub‐microsecond switching, endurance beyond 10^8^ cycles, and array‐level (3 × 3) demonstrations of parallel inference, weight‐update operations, and XOR classification (Figure 7c) [38]. More recently, Wang and colleagues reported a vertical OECT‐based memory capable of 10‐bit non‐volatile states and wide dynamic range of 32 (Figure 2c) [74]. Notably, the device exhibited excellent stability, maintaining retention for up to 10,000 s in ambient air with the gate grounded, and demonstrated a low conductance drift coefficient between 0.003 and 0.006. Furthermore, hardware ANNs based on organic device arrays have also been integrated into a neuromorphic biosensing platform with on‐chip learning ability, as exemplified by the classification of the genetic disease cystic fibrosis from modified donor sweat [165]. A comparison of representative state‐of‐the‐art transistor‐based organic non‐volatile memories is summarized in Table 2. While notable improvements have been achieved in key metrics such as retention time, dynamic range, and write–read cycle stability, large‐scale array demonstrations remain limited. Moreover, it is important to note that reported performance metrics are often achieved under separate conditions rather than simultaneously in a single device.

**TABLE 2:** Summary of state‐of‐the‐art organic transistor‐based organic non‐volatile memory.

Year	Channel Material	Electrolyte/ Gate Dielectric	Retention/ Related Number of States	Dynamic Range	Write Pulse Width/ Write‐read Cycle	Number of States	Array Size	Origin of Non‐volatility	Reference
2017	PEI/PEDOT: PSS	Nafion	>100 s/ 5 >25 hr/ 1	∼1.2	6 ms/ ‐	>500	—	ECRAM	[37]
2019	PEI/PEDOT: PSS	Nafion	>10 min/ ‐	<2	200 ns/ 1 µs	>50	3 × 3	ECRAM	[38]
2020	P(g2T‐TT)	Ion gel	>5 min/ 3	>2	20 ns/ <1 µs	>100	—	ECRAM	[83]
2022	ETE‐PC	NaCl aqueous solution	>1000 s/ 2	∼2	1 s/ ∼10 s	>150	—	Electro‐polymerization	[89]
2022	PIBET‐A	Ion gel	>5000 s/ 7	∼66	40 ms/ ‐	>200	—	Ion Trapping	[95]
2022	P(gT2)	Organo‐hydro‐gel	>10^4^ s/ 2	>100	500 µs/ ‐	>800	3 × 3	Gate‐channel Open Circuit	[166]
2023	PTBT‐p	Ion gel	>10^4^ s/ 6	32	200 ns/ >1µs	>1000	9 × 2	Ion Trapping	[74]
2024	gNR‐Pr	Gel electrolyte	>800 s/ 4	∼147	0.26 s/ ‐	>256	—	Ion Trapping	[96]
2025	PEDOT:PSS	Solid electrolyte	>100 s/ 12	∼110	700 µs/ >1.1 ms	>50	—	Ion Trapping	[167]

While hardware‐based ANNs have achieved remarkable progress, they still rely on real‐valued computations and synchronous updates, which diverge from the sparse, event‐driven nature of biological information processing. Spiking neural networks (SNNs) aim to bridge this gap by encoding and transmitting information through discrete spikes, where processing is governed by the timing of these events [168]. This temporal coding paradigm not only provides a closer analogy to neuronal signaling but also promises substantial gains in energy efficiency by activating computation only when spikes occur. Furthermore, SNNs naturally integrate with local learning rules such as STDP, enabling online and unsupervised learning in dynamic environments [169].

However, despite the impressive progress in developing organic artificial neurons and numerous demonstrations of STDP with organic neuromorphic electronics, reports of organic hardware‐based SNNs remain scarce. Wang et al. developed nine cv‐OECT‐based STDP synapses in a 1T‐1R architecture [74]. Simulation of a single‐layer SNN achieved ∼89% accuracy on MNIST classification, comparable to the cv‐OECT‐based ANN (∼91%). More recently, Zhu et al. designed a LIF‐type artificial neuron based on OECTs, capable of processing temporal‐coded information [170]. Using this LIF neuron, a SNN with 10 input nodes was simulated to recognize handwritten Chinese characters encoded into spike trains. Employing a precise‐spike‐driven (PSD) supervised learning rule, the network successfully learned to fire spikes at desired times. During recognition, the output class was determined by the neuron with the highest firing frequency, achieving up to 98.5% accuracy even for noisy datasets containing two Chinese characters. Although elementary in scale and function, current demonstrations of organic hardware‐based SNNs highlight the feasibility of spike‐based temporal coding in organic platforms and pave the way toward more complex, energy‐efficient, and biologically realistic neuromorphic systems.

#### 6.1.2 Physical Reservoir in Reservoir Computing

Reservoir computing (RC) is a computational framework composed of a fixed dynamic reservoir which projects input signals into a high‐dimensional state space via non‐linear transformation, and a simple readout layer, typically implemented through linear regression or classification, for pattern analysis [171]. Originating from recurrent neural networks (RNNs), RC exhibits strong capabilities in processing temporal and sequential information. Importantly, because only the readout weights require training, RC significantly reduces the training complexity and computational cost compared to conventional neural networks [172]. Moreover, as the reservoir itself does not require adaptive updates, hardware‐based implementation of RC is highly feasible and particularly well‐suited for in‐sensor computing paradigms. In this section, we review the physical realization of reservoirs using organic neuromorphic electronics, while related in‐sensor computing applications are discussed in subsequent sections.

Memristive materials and devices have been widely explored as physical nodes for RC owing to their ability to exhibit the two properties essential for effective reservoir operation: nonlinear input–output signal transformation and short‐term, input‐history–dependent responses, which underpin the state richness and memory capacity of the reservoir [171, 173, 174]. Depending on how these properties are harnessed, two general architectures have emerged: in materia RC and dynamic devices RC [174]. In in materia RC, the intrinsic dynamics of the material provide high‐dimensional mapping, demonstrating spatially heterogeneous conductivity between input and output terminals so that signals undergo distinct nonlinear transformations. By contrast, dynamic devices RC constructs the reservoir from an ensemble of individual devices, each providing nonlinear dynamics and volatile memory. When driven in parallel, these devices collectively generate a large number of effective physical nodes, thereby improving the system performance.

Among them, organic artificial synapses fabricated through bottom‐up approaches such as the electropolymerization of conductive polymers have shown particular promise for constructing nonlinear random neural networks as physical reservoirs in in materia RC [82, 174, 175, 176, 177], with potential applications in monitoring and analyzing biological activities. A notable example was reported by Cucchi et al., who demonstrated a biocompatible RC system based on OECTs for real‐time bio‐signal classification [175]. In their work, dendritic networks of PEDOT doped with hexafluorophosphate (PEDOT:PF_6_) were stochastically grown through AC polymerization across multiple metal pads, forming semirandom OECT channels within a shared electrolyte (Figure 7d). By maintaining a balance between excitatory and inhibitory fibers and leveraging the global electrolyte‐gating effect characteristic of OECTs [178], strong nonlinear transformations of the input signals was achieved through the reservoir. Fourier analysis of sinusoidal inputs confirmed the emergence of new frequency components, indicating effective nonlinear projection and good separability of input states. When coupled with an analog delay‐line feedback loop, the OECT‐based reservoir achieved an overall classification accuracy of 88% for four classes of arrhythmic heartbeat signals from the MIT‐BIH dataset.

Photonic dynamic devices RC has also been realized using organic artificial synapses. Wu and colleagues developed a photo‐responsive wearable in‐sensor RC system designed for multi‐tasked pattern classification [179]. At the material level, they developed a bottlebrush‐shaped semiconducting polymer (*p*‐NDI) with efficient exciton dissociation and slow charge recombination, enabling key reservoir properties such as excellent separability, fading memory, and echo state behavior. Transistors based on *p*‐NDI exhibited 32 distinguishable states under different input optical pulse trains (Figure 7e), reflecting robust nonlinear dynamic evolution. By constructing a light‐responsive reservoir circuit with multiple *p*‐NDI synaptic transistors, the system could extract visual features from image inputs. Coupled with a memristive organic diode crossbar array as the readout layer, the fully organic optoelectronic RC platform achieved multi‐task learning with high efficiency. Specifically, it attained an overall garment‐type and size recognition accuracy of 88.00%, which is comparable to that of single‐ (92.40%) and double‐layer ANNs (94.51%). Remarkably, the accuracy per weight was 0.033 for the RC system, far surpassing ANN counterparts (0.0073 for SL‐ANN and 0.00089 for DL‐ANN), highlighting its much lower training cost. Beyond static images, the RC system also processed event‐based video inputs, achieving 98.62% accuracy in classifying three types of hand gestures. Furthermore, the number of multiplication‐and‐accumulation operations required during training and inference was reduced by more than an order of magnitude, underscoring the efficiency of the system for edge learning applications. Similar physical reservoir configurations have also been employed in electrically gated OECT‐based RC systems [96, 167, 180].

More recently, Chen and colleagues reported an ultra‐short channel organic neuromorphic vertical field effect transistor designed with distributed reservoir states (VOFET‐DR) for implementing grouped RC networks [181]. The device employed a BHJ channel of N2200: P0FDIID, an Al_2_O_3_(1 nm)/polyvinyl alcohol (PVA) charge trapping layer, and a MXene thin film source electrode (Figure 7f). The OFET exhibited short‐term memory characteristics in response to both gate‐voltage and light pulses, enabling its use as a physical node for RC. Under light‐pulse sequences, the device reproducibly exhibited 64 distinct conductivity states, effectively mapping nonlinear temporal characteristics into the reservoir space. To realize grouped RC, distributed reservoir states were further enriched by applying different gate biases during light‐pulse stimulation, generating a total of 1152 reservoir states. As a proof of concept, four sub‐reservoirs of 100 physical nodes each, biased at different gate voltages, were grouped in parallel to classify complex features in satellite remote‐sensing images. The system achieved feature‐recognition accuracy exceeding 95%, comparable to conventional ANN (96.1%) and CNN (92.1%) benchmarks, while reducing weight‐related computational costs by more than 90%.

### 6.2 Multimodal Sensory Systems

#### 6.2.1 In‐Sensor Computing

With the rapid proliferation of sensory nodes and the accompanying explosion of raw, unstructured sensing data, conventional computing architectures face critical challenges. Continuous data conversion and transfer between separate sensing, memory, and processing units—particularly for redundant data—introduce significant latency, consume excessive communication bandwidth, increase energy costs, and often degrade data precision. To address these limitations, local processing paradigms such as near‐sensor and in‐sensor computing have been proposed [140, 182]. By integrating sensing and processing functions close to or directly within the sensory nodes, these approaches relocate computational tasks closer to the data source, thereby alleviating transfer bottlenecks and enhancing overall energy efficiency.

Within this context, organic artificial synapses have emerged as compelling candidates for in‐sensor computing. Their intrinsic sensing adaptability—manifested through excitatory and inhibitory responses that depend on stimulus width, number, frequency, and physical properties (e.g. light intensity and wavelength)—maps directly onto key computational primitives for low‐level signal processing, enabling noise suppression, background extraction, feature enhancement, and motion extraction to be performed directly at the sensory interface. Moreover, when sensory synaptic devices exhibit properties compatible with hardware ANNs or physical RC systems, they can also support high‐level sensory processing driven by non‐electrical inputs. Paired with the mechanical softness and biocompatibility of organic materials, these attributes position organic synaptic devices as highly promising building blocks for next‐generation wearable electronics and bio‐integrated sensing platforms. In the following, we highlight representative examples that demonstrate these capabilities.

#### 6.2.2 In‐Sensor Computing of Optical Signals

Vision is the dominant human sense, with more than 80% of external information perceived through visual pathways [117]. Emulating this capability has therefore been a central research focus. Compared with conventional artificial vision systems, emerging neuromorphic visual platforms based on optoelectronic artificial synapses integrate sensing, memory, and processing of optical signals within a single device. This enables in situ image (pre‐)processing with substantial advantages in energy efficiency and circuit simplicity [30, 33, 183]. Owing to their easily tunable light absorption range, solution processability, low‐cost fabrication, and biocompatibility [8, 130, 184, 185], organic active materials have been widely adopted for the development of photonic artificial synapses. These devices have demonstrated functionality across multiple processing levels in in‐sensor computing: from low‐level operations such as noise filtering and feature enhancement to higher‐level tasks involving abstraction and pattern recognition.

**FIGURE 8:**
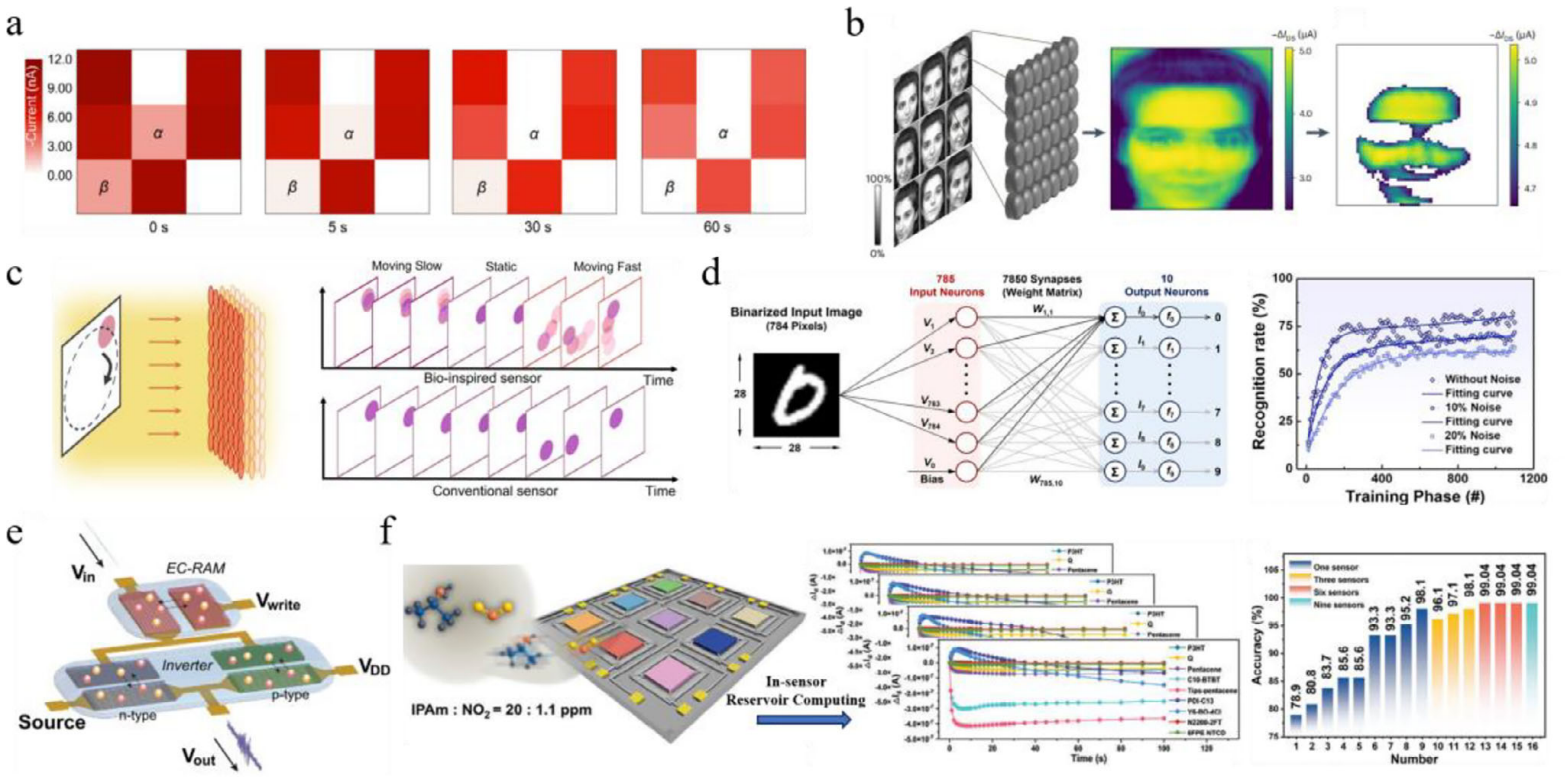
a) Visual noise reduction simulated in a 3 × 3 OSP array with “V” as the test pattern. Reproduced with permission [186]. Copyright 2025, American Chemical Society. b) Training process of an artificial retina generating a memory current mapping of a women face. Reproduced with permission [115]. Copyright 2023, Springer Nature. c) Motion video of a moving ball captured by a sensor array, demonstrating bioinspired extraction of temporal and spatial information. Reproduced with permission [114]. Copyright 2025, Springer Nature. d) Schematic of a SLP‐based ANN and recognition rate as a function of training phase. Adapted with permission [187]. Copyright 2022, Springer Nature. e) Schematic of an adaptable inverter. Reproduced with permission [84]. Copyright 2022, Wiley‐VCH. f) Schematic of the gas sensor array, representative response patterns of individual sensors to different gases, and classification accuracies of 16 sensor‐arrays with varying number of sensors. Adapted with permission [190]. Copyright 2025, Wiley‐VCH.

As an example of low‐level in‐sensor computing, Guo et al. developed an organic synaptic phototransistors (OSP) based on a semiconductor heterojunction comprising the p‐type poly(2,5‐bis(2‐octyldodecyl)‐3,4‐dicyanothiophene) PDPP4T and the n‐type NTCDI‐F15 [186]. The device exhibited typical synaptic behaviors under optical stimulation, including EPSC, PPF, and transitions from STP to LTP depending on the number, width, intensity, and frequency of light pulses. To demonstrate in‐sensor noise reduction, a 3 × 3 OSP array was fabricated and irradiated with a letter ‘V’ encoded by ten continuous light pulses superimposed with noise induced by a single light pulse at other pixels. With its light‐adjustable synaptic behaviors, after 1 min of attenuation, the noise information decayed and became indistinguishable, whereas the letter ‘V’ was effectively retained, mimicking the noise‐filtering function of the visual neural system (Figure 8a).

Mei and colleagues reported an organic optoelectronic synapse that incorporates a photoactive donor–acceptor BHJ layer into the channel of OECTs, enabling photon‐modulated electrochemical doping for in‐sensor visual processing [115]. The device exhibited both STP and LTP which depended on light pulse properties, arising from light‐driven ion insertion into the channel. To demonstrate system‐level functionality, the authors simulated facial recognition using a 64 × 64 optical synaptic array (Figure 8b). When nine greyscale face images of a woman with distinct expressions and orientations were input to the array, regions with higher optical reflectivity produced stronger memory currents due to the device's synaptic behaviors, allowing the extraction of high‐level facial features. With appropriate decision‐making criteria, the artificial retina achieved successful recognition of target faces from four test images, illustrating the potential of such organic optoelectronic synapses for bioinspired vision systems.

Motion perception has also been demonstrated in optical in‐sensor computing applications. Wang et al. developed an organic optoelectrochemical synapse incorporating an n‐type OMIEC film within a micron‐scale channel operated in aqueous electrolyte [114]. The channel was based on an all–electron‐accepting copolymer, p(C2F‐z), composed of fluorinated bisisatin‐lactone and bithiazole units, which responded to both optical and electrical stimuli. Owing to its broad absorption spectrum, the synaptic transistor exhibited short‐term plasticity under optical excitation across the UV, visible (VIS), and near‐infrared (NIR) ranges, attributed to light‐induced aqueous ion doping of the channel. To demonstrate system‐level functionality, the authors fabricated an 8 × 8 OECT array which not only exhibited image memorization but also enabled motion perception through the device's memory function. In this task, sequential optical stimuli generated by moving balls were projected onto the array, and the synaptic responses encoded both spatial information (object position) and temporal dynamics (motion trajectory) (Figure 8c). These spatiotemporal features were then mapped into dynamic current patterns, forming a feature map that effectively represented the direction and velocity of motion. When coupled with an ANN for classification, the system achieved a recognition accuracy of 92.5% in distinguishing motion direction and speed, highlighting its capability for dynamic visual perception.

A widely adopted strategy for high‐level in‐sensor computing with optoelectronic artificial synapses is the construction of reconfigurable device arrays for neural network computations directly at the sensory level. The synaptic and device‐level requirements largely mirror those of hardware ANN synapses—such as linear and symmetric conductance updates, a large number of non‐volatile states, and an adequate dynamic range—with the key distinction that optical stimuli, rather than electrical signals, serve as the primary inputs driving synaptic responses. While the detailed operation principles of such arrays and their associated algorithms have been reviewed elsewhere [33, 140], we therefore only provide a brief overview here, highlighting representative implementations realized using organic synaptic devices. For example, Zhang and co‐workers developed an organic optoelectronic synaptic transistor employing wood‐derived cellulose nanopaper as both dielectric and substrate, combined with a PDPP4T/chlorophyll‐a hybrid film as the photoactive layer. Using this device, a maximum recognition rate of ∼79% was achieved for MNIST handwritten digit classification, based on the simulation of a single‐layer perceptron after 800 training epochs (Figure 8d) [187]. Similarly, Mei et al. simulated both a single‐layer perceptron and a deep LeNet convolutional neural network (CNN) using their optical synapse, achieving recognition rates of 69.0% and 89.5% on the Fashion‐MNIST dataset, respectively [115].

#### 6.2.3 In‐Sensor Computing of Biological Signals

Organic devices offer significant potential as biosensors due to their biocompatibility, simple fabrication, flexibility, stretchability, and energy efficiency [41, 128, 130, 188]. By integrating these advantages with synaptic functionality, organic neuromorphic biosensors can be envisioned as hardware platforms for intelligent tasks such as drug delivery, prosthetic motion control, and health monitoring [189]. Recently, Zhang et al. reported an adaptive organic complementary logic inverter capable of in‐sensor processing and tailoring of biologically relevant signals, including electromyograms (EMGs) and electrocardiograms (ECGs) [84]. The authors developed an ambipolar polymer, P‐3O, which was used to construct both the complementary inverter for signal amplification and normalization, and a non‐volatile artificial synaptic device to locally tune the gain. As shown in Figure 8e, the inverter was composed of two complementary organic transistors operating in p‐ and n‐type modes, while the artificial synapse, based on an ECRAM structure, was placed at the gate terminal. By modulating the conductance of the ECRAM, the inverter exhibited locally and directly tunable amplification, enabling adaptive adjustment of the output in response to varying biosignal amplitudes. Leveraging this functionality, the authors demonstrated that the signal envelopes of EMG recordings from two distinct hand gestures could be accurately preserved after tuning the inverter gain to appropriate states.

Reservoir computing has been applied to in‐sensor processing of biological signals. Liu et al. reported a wearable in‐sensor computing platform based on intrinsically stretchable OECT (ISOECT) arrays which exhibited over 50% stretchability [180]. The nonlinearity of the ISOECTs arose from the operational dynamics of the OECTs, enabling them to produce 16 distinguishable states in response to sequential input signals, thereby making them well‐suited for RC. A 4 × 4 ISOCET RC array was subsequently fabricated and integrated with an ISOECT sensor array and a coin‐sized readout unit, forming a wearable integrated soft electronic (WISE) platform. In operation, EMG signals were detected and amplified by the sensor array, encoded into four‐bit sequential voltage pulse streams by the readout unit, and then processed by the RC array, enabling accurate recognition of three distinct hand gestures.

#### 6.2.4 In‐Sensor Computing of Chemical Signals

Reservoir computing has also been applied to in‐sensor processing of chemical signals. Wu and colleagues integrated an OFET array integrated with in‐sensor RC and a k‐nearest neighbors (KNN) algorithm for gas classification [190]. The multi‐OFET array incorporated nine different semiconductors: P3HT, quinacridone (Q), pentacene, C10‐BTBT, TIPS‐pentacene, PDI‐C13, Y6‐BO‐4Cl, N2200‐2F, and 5FPE‐NTCDI, providing diverse gas responses (Figure 8f). Each OFET exhibited distinct nonlinear drain‐current behaviors, varying in magnitude and direction over time, when exposed to eight different analytes. Collectively, these temporal response patterns constructed unique feature signatures for each gas. The OFET array thus served as a physical reservoir, with three‐dimensional output features defined by current magnitudes and directions sampled at five time points. These features were subsequently classified using a KNN algorithm. The 3 × 3 OFET RC system achieved 100% accuracy in distinguishing eight volatile compounds (methanol, acetone, ethyl ether, toluene, isopropylamine, DMMP, NO_2_, and nitrobenzene). Furthermore, by expanding the temporal sampling to 32 points, the system reached 99.04% accuracy in identifying a library of 26 analytes, including single gases, isomers, homologs, and mixtures.

#### 6.2.5 Multisensory Integration

Biological systems benefit greatly from their ability to integrate multiple sensory inputs, enabling more accurate perception and optimal decision‐making. In the previous section, we reviewed advances in synaptic multimodal sensing functions achieved at the single‐device level using organic artificial synapses. Here, we shift the focus to applications of MSI in organic neuromorphic electronics, encompassing both device‐ and circuit‐level implementations, and addressing the fusion of multiple inputs of the same modality as well as cross‐modal signals.

**FIGURE 9:**
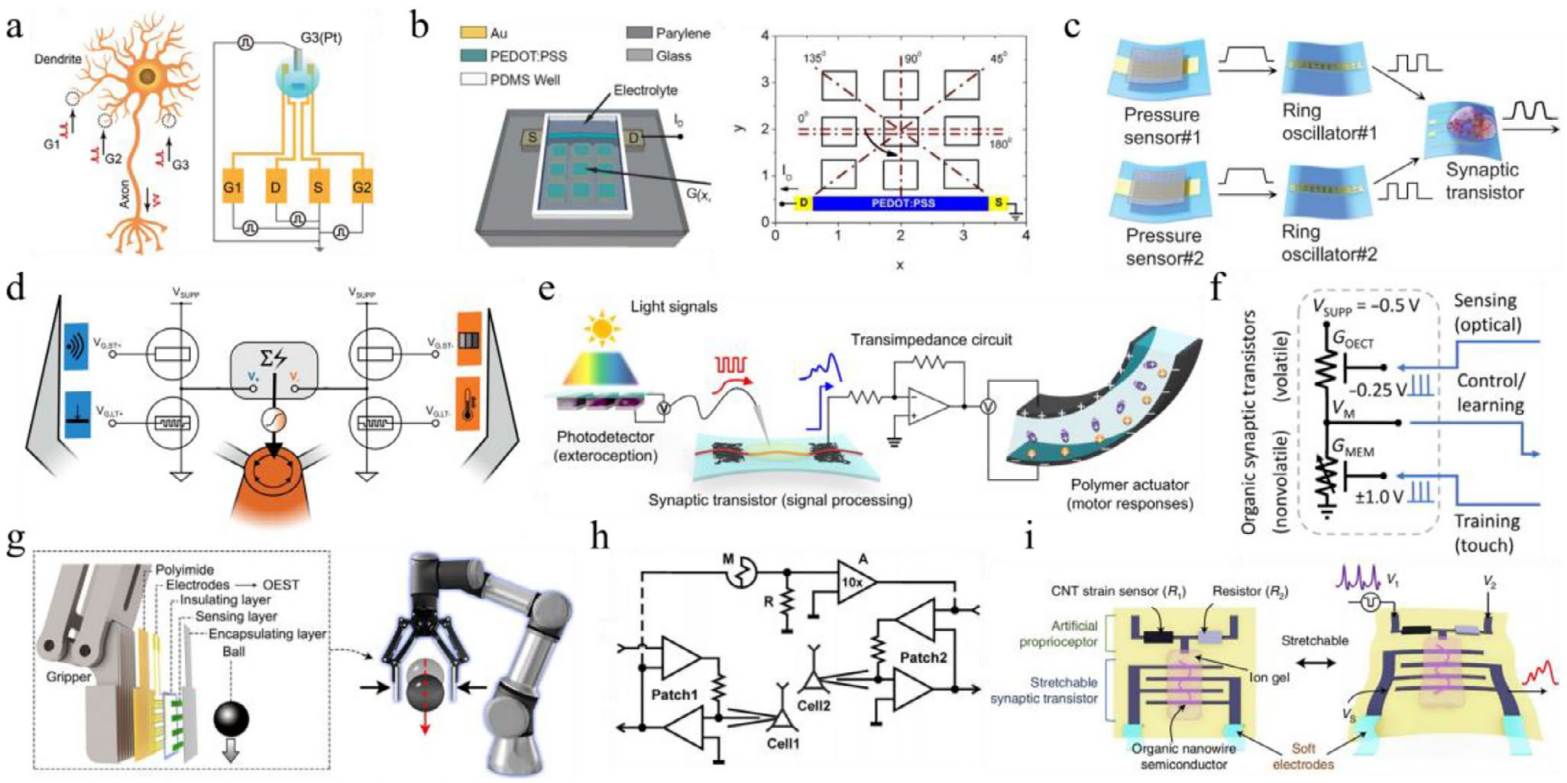
a) Solution‐gated OECT with two in‐plane Au gates (G1, G2) and one Pt gate (G3) for spatial signal summation and its biological analogy. Reproduced with permission [39]. Copyright 2019, WILEY‐VCH. b) Multi‐gate single‐channel OECT with orientation selectivity and definition of spatial pulse patterns with different orientations. Adapted with permission [192]. Copyright 2016, Springer Nature. c) Artificial afferent nerve with two branches of ring oscillators and pressure sensors. Reproduced with permission [121]. Copyright 2018, AAAS. d) Organic neuromorphic circuit with a two‐branch (+/–) architecture. Adapted with permission  [142]. Copyright 2024, Springer Nature. e) Organic optoelectronic sensorimotor synapse comprising an organic optoelectronic synapse and a neuromuscular system. Reproduced with permission [194]. Copyright 2018, AAAS. f) Schematic of the organic neuromorphic circuit. Adapted with permission [195]. Copyright 2021, AAAS. g) AOAN integrated on a robotic gripper and demonstration of slip detection with a metal ball. Reproduced with permission [123]. Copyright 2024, Springer Nature. h) Simplified equivalent circuit of an OMD‐based neural interface connecting two neurons. Reproduced with permission [197]. Copyright 2018, WILEY‐VCH. i) Artificial neuron integrating a DA electrochemical sensor, memristor‐based artificial synaptic device, and DA‐encapsulated heat‐responsive hydrogel. Reproduced with permission [198]. Copyright 2022, Springer Nature.

Dendritic integration plays a critical role in temporal and spatial signal processing in the brain [9, 191]. Inspired by this mechanism, a multi‐gate single‐channel organic artificial synapse (Figure 9a) was designed to emulate biological signal summation by exploiting the spatiotemporal capacitance coupling mediated by the global electrolyte and demonstrated basic spatiotemporal‐correlated logic operations such as “AND”, “OR”, and “YES_G3_” [39]. Similarly, spatial orientation selectivity was realized in a multi‐gated PEDOT:PSS OECT (Figure 9b) [192]. Beyond these elementary operations, dendritic integration has also enabled more complex near‐sensor computing applications. For example, Kim and co‐workers reported an artificial afferent nerve by integrating resistive pressure sensors, organic ring oscillators, and a synaptic transistor [121]. Pressure inputs collected by the sensor array were converted into voltage pulses through the ring oscillators and subsequently integrated by the synaptic transistor through multiple gate electrodes. The postsynaptic output exhibited additive properties in both time and frequency domains, confirming effective spiking signal integration (Figure 9c). Leveraging this architecture, the system successfully identified Braille characters and emulated biological reflex arcs using single or multiple artificial afferent nerves. Compared with conventional silicon‐based synaptic circuits, the organic artificial synapse enabled simpler circuit design and enhanced feature discrimination in Braille recognition, highlighting its potential for advanced feature extraction and signal integration.

Beyond predefined MSI functions, Krauhausen et al. demonstrated a multimodal real‐time learning process using organic neuromorphic electronics in a robotic manipulator interacting with its environment [142]. The system integrated volatile OECTs with non‐volatile ECRAMs to form a neuromorphic circuit capable of helping distinguish safety from potentially harmful objects for robotics. The robotic manipulator, driven by an Arduino Braccio Kit, was equipped with four sensors responsive to pressure, distance, temperature, and color tone, and could perform pick‐up, drop, or pull‐back actions. The circuit comprised two voltage dividers, each consisting of an OECT and an ECRAM in series, functionally resembling dendritic summation (Figure 9d). Associative adaptation emerged from the interplay between OECT volatility and ECRAM memory, with the combined output voltage determining the probability of a given motor action. Learning proceeded in stages. Initially, the manipulator acted randomly, grabbing or not grabbing a cup independent of stimuli. In the first cycle, proximity and pressure sensors were connected to the gates of the OECT and ECRAM in the V^+^ branch. Successful grasping triggered pressure feedback that increased the branch output, establishing an associative link between proximity and the grabbing action. In a second stage, thermal input, correlated with cup color, was applied to the ECRAM in the V^−^ branch, modulating its output and establishing an association between temperature and color. When combined with the V^+^ branch, the summed output ΣV reached high intensity in response to hot, dark cups, which the system interpreted as noxious, prompting a protective withdrawal. This demonstrated multimodal sensory learning and behavioral conditioning through organic neuromorphic integration.

#### 6.2.6 Sensorimotor Systems

With sensing, memory, and signal‐processing capabilities, artificial synapses can be integrated with motor units to emulate the neural control of movement and exhibit neuromorphic behavioral conditioning responses [58, 193]. Such neuromorphic sensorimotor systems hold promises for bioinspired robotics, electronic prosthetics, and exoskeletons capable of natural and adaptive motion. Sensorimotor integration—the simplest form of interaction achieved through the biological reflex arc—directly links sensory input to motor output, thereby enhancing response speed and reducing computational overhead, and has thus become an active research focus [45].

As an illustrative example, Lee et al. developed an organic optoelectronic sensorimotor artificial synapse by integrating a photodetector with a stretchable organic nanowire synaptic transistor (s‐ONWST) to drive an artificial muscle actuator (Figure 9e) [194]. The s‐ONWST was fabricated with an FT4‐DPP:PEO nanowire channel, a styrene–ethylene–butylene–styrene (SEBS) substrate, carbon nanotube source/drain electrodes, and an ion gel electrolyte. Coupled with the photodetector, the device functioned as an optoelectronic artificial synapse exhibiting synaptic plasticity in response to light stimuli. It could convert patterned optical signals, such as International Morse code, into distinct postsynaptic currents, thereby demonstrating optical wireless communication. When connected to a polymer actuator through a transimpedance circuit, the postsynaptic currents generated by the optoelectronic synapse were converted into voltage signals capable of driving the actuator, yielding significantly larger displacements compared to direct coupling. Through this configuration, the sensorimotor synapse enabled remote optical control actuation of artificial muscle contraction and effectively emulated biological muscle tension response to APs.

Another example of sensorimotor integration, which highlights decentralized and distributed behavioral learning, was recently demonstrated by Gkoupidenis and co‐workers. They designed an adaptable neuromorphic circuit based on organic artificial synapses which enabled a robotic system to learn and navigate through a maze [195]. The circuit functioned as a trainable voltage divider composed of a non‐volatile organic memristor (MEM) and a volatile OECT, with the output voltage V_M_ determining the robot's turning direction at maze intersections. Mechanical and optical sensory signals were mapped onto the gates of the MEM and OECT, respectively. As illustrated in Figure 9f, the visuomotor association learning process relied on the complementary roles of the two devices: mechanical inputs provided training pulses that gradually tuned the conductance of the MEM, setting the baseline of V_M_, while optical inputs transiently modulated the OECT, biasing V_M_ trigger turning decisions. Through reinforcement by repeated mechanical stimuli—delivered either by an external trainer or when the robot collided with maze boundaries—the system established a stable visuomotor association. Once trained, the robot reliably turned left in response to visual cues and right in their absence, enabling it to autonomously navigate out of the maze with visual guidance.

Tactile sensing has also been integrated with motor output to demonstrate the sensorimotor loop [121, 196]. In a recent study, Chen and colleagues reported a flexible artificial organic afferent nerve (AOAN) by coupling pressure‐sensitive mechanoreceptors with an OECT‐based synaptic transistor (channel: PFT‐100; electrolyte: ion gel) [123]. The integrated AOAN device exhibited both STP and LTP in response to pressure stimuli and, when four mechanoreceptors arranged in line were connected in parallel to the gate of the synaptic OECT, enabled spatiotemporal motion detection. Asynchronous activation of the mechanoreceptors produced a stronger postsynaptic response than either independent or synchronous activation, owing to post‐tetanic potentiation in the AOAN. This enhanced response served as a clear indicator of sliding motion. Integrated into a robotic gripper, the AOAN system could detect slip events in real time through spike‐feature extraction and, via a closed‐loop control algorithm, trigger adaptive slip‐prevention actions (Figure 9g). As a result, the gripper autonomously adjusted its grasp to maintain a secure hold without external intervention. Notably, during rapid shaking, the intelligent gripper successfully prevented a ball from slipping by dynamically modulating its grip in response to tactile feedback, demonstrating robust real‐time sensorimotor integration.

### 6.3 Neuromorphic Bio‐Integrated Interfaces

Alongside neuromorphic computing and sensing, an intuitive application of artificial synapses is to replicate the function of biological synapses in modulating and relaying neural signals, thereby establishing functional interfaces with living neural networks for prosthetic devices or biohybrid systems. To date, such neuromorphic bio‐integrated interfacing has been demonstrated using both electrical and chemical outputs from, as well as inputs to, living cells. For instance, Juzekaeva et al. reported the first experimental evidence of functional coupling between live cortical neurons using an organic memristive device (OMD) based on polyaniline (PANI) and lithium salt doped PEO as the solid electrolyte (Figure 9g) [197]. In their setup, two naturally unconnected cortical pyramidal neurons in rat brain slices were linked by an electronic circuit incorporating a single OMD as the synapse analog. Initially, the OMD was conditioned to a high‐resistance state, so that APs in the “presynaptic” Cell 1 could only evoke subthreshold depolarizations in the “postsynaptic” Cell 2. With repeated stimulation of Cell 1, the OMD resistance gradually decreased, enhancing signal transmission. Once the resistance dropped to about half its initial value, Cell 2 reliably fired APs, indicating successful activity‐dependent spike coupling mediated by the OMD. Moreover, the OMD‐based synapse reproduced key temporal features of biological synapses, such as STDP, and it enabled synchronous delta oscillations in the two‐neuron network. These results provided the first demonstration of unidirectional, activity‐dependent coupling of living neurons through an organic artificial synapse, highlighting its potential for prosthetic synapses and hybrid neuromorphic systems.

In another example, Lee et al. described a stretchable neuromorphic efferent nerve (SNEN) which bypassed damaged electrophysiological signal pathways and delivered neuromorphic electrical signals to muscles for controlled movement [199]. The SNEN integrated three components: (i) a carbon nanotube (CNT) strain sensor acting as an artificial muscle spindle to detect leg extension, (ii) a stretchable synaptic transistor composed of fused thiophene diketopyrrolopyrrole (FT4‐DPP)–based conjugated polymer nanowires as the semiconducting channel and an ion gel poly(styrene‐b‐methyl methacrylate‐b‐styrene) (PS‐PMMA‐PS) triblock copolymer with EMIM:TFSI as the gate dielectric, and (iii) soft hydrogel electrodes for compliant electrical interfacing. The synaptic transistor exhibited activity‐dependent signal potentiation in response to presynaptic APs applied at the gate, which translated into gradually increased muscle force and smooth hind‐limb motion when connected to a mouse leg. Meanwhile, the CNT strain sensor provided proprioceptive feedback by modulating its resistance with muscle extension, thereby regulating the input to the synaptic transistor and forming a closed feedback loop that prevented overstraining. To highlight its functional potential, the SNEN was attached to the leg of a mouse, enabling locomotor tasks including ball kicking, bipedal walking, and running through controlled AP inputs.

As for chemical signals, Keene and colleagues demonstrated a neurotransmitter‐mediated organic artificial synapse capable of both sensing and long‐term memory in response to DA signals at a presynaptic electrode (Figure 5c) [133]. Building on this concept, Wang et al. recently developed a chemically mediated artificial neuron that integrates a DA electrochemical sensor, a memristor‐based artificial synapse, and a DA‐encapsulated heat‐responsive hydrogel (Figure 9i) [198]. In this system, the electrochemical sensor transduced extracellular DA into electrical signals that stimulated the memristor, producing STP and LTP behaviors characteristic of a neurotransmitter‐mediated artificial synapse. When connected in series with a microheater covered by the DA‐loaded hydrogel, the memristor also regulated neurotransmitter release, enabling controllable DA secretion. Importantly, the artificial neuron exhibited adaptive behavior: repeated DA stimuli drove the memristor into a low‐resistance state, thereby lowering the threshold required for subsequent DA release, akin to synaptic strengthening in biological neurons. To emulate interneuronal communication, the device was interfaced with living neuronal cells. A clear current response was observed when cell‐incubated PBS was delivered across the sensor, demonstrating reception of chemical signals from cells. Conversely, DA released from the hydrogel at concentrations of ∼100 µM successfully activated PC12 cells, confirming bidirectional chemical communication at the biohybrid interface.

## 7 Conclusion

Recent advances in neuromorphic electronics have opened a new frontier for organic electronics. Leveraging low operating voltage, synthetic tunability, mechanical softness and biocompatibility, organic platforms now support neuromorphic functionalities that extend well beyond rudimentary synaptic emulation. Organic artificial neurons reproduce diverse spiking dynamics and interface directly with biological systems, enabling event‐driven sensing. Organic synaptic transistors implemented in crossbar arrays for ANN acceleration demonstrated low switching energies, large numbers of analog states, and favorable state linearity and repeatability, collectively enabling near‐ideal accuracy in neural network simulations. In the context of in‐sensor computing, organic synapses can combine sensing, memory and processing in a single element and fuse multimodal inputs, underscoring their potential for all‐organic edge computing. Early demonstrations of sensorimotor integration illustrate that organic systems can co‐ordinate sensing, memory, learning and actuation to produce fast, low‐power, adaptive behaviors—capabilities that point toward applications in soft robotics, exoskeletons, neuroprosthetics and smart drug delivery. Finally, as neuromorphic bio‐interfaces, organic devices offer distinct advantages: their biocompatibility enables direct communication with living cells and tissues, while their ability to emulate STP, LTP, and spike‐dependent plasticity supports adaptive, bio‐inspired information processing. The combination of soft mechanics, biochemical sensitivity and facile biological integration positions organic neuromorphic electronics as promising candidates for wearable and implantable bioelectronics.

Nevertheless, organic neuromorphic electronics remain at an early, largely laboratory‐scale stage and several substantive challenges must be addressed before practical deployment. At the materials level, stability and reliability are primary concerns. OMIECs operating in biological or ambient environments inevitably encounter oxygen, water, and reactive molecular species. For p‐type OMIECs, spontaneous oxygen doping and side reactions generate undesirable byproducts that deteriorate electronic performance and accelerate degradation. Strategies such as lowering the HOMO level (≤ −4.9 eV), incorporating electron‐accepting comonomers, or improving polymer purity can mitigate these effects [200]. n‐Type OMIECs suffer from severe instability due to oxygen‐reduction reactions (ORR), leading to poor ambient performance and hindering the development of complementary organic circuits [40, 130, 200]. Achieving a deep LUMO is essential for stabilizing electron transport in air, yet designing air‐stable, high‐performance n‐type OMIECs remains a persistent and unresolved challenge [44]. Meanwhile, many semiconducting polymers also experience moisture‐driven degradation, where absorbed water forms trap states that compromise charge transport [201, 202]. Encapsulation can mitigate oxygen and moisture ingress, but often increases fabrication complexity and may reduce mechanical compliance in wearable or bio‐integrated systems. Although elastomers such as butyl rubber are commonly used, their barrier performance remains far inferior to metals or inorganic oxides [203], underscoring the need for encapsulation materials that combine high stretchability with ultralow air and water permeability. Mechanical and interfacial stability present additional barriers. Ion‐driven volumetric swelling during doping cycles can disrupt polymer microstructure, degrade mobility, and accelerate mechanical fatigue [204, 205, 206]. Excess swelling can be alleviated through molecular engineering, such as introducing hydrophobic comonomer or incorporating structural spacers [200]. In wearable and implantable systems, repeated bending, stretching, and twisting further promote cracking and delamination, making mechanical compliance and modulus matching with soft tissues essential [130, 203, 207, 208]. Despite recent advances in intrinsically stretchable OMIECs and device architectures [130, 207], device failure often originates at weak or poorly engineered interfaces during cyclic deformation. Interfacial engineering strategies, such as chemical functionalization and the use of adhesive interlayers, offer promising routes to enhance adhesion and improve long‐term mechanical stability [207, 209].

Emerging material concepts offer additional opportunities. Self‐healing materials, defined by their ability to autonomously repair physical damage [210], can substantially extend device lifetime, particularly in flexible, stretchable or bio‐integrated systems where tearing, microcracking, and repeated mechanical deformation are common. In most reported materials, healing arises from reversible dynamic covalent bonds (e.g., Diels–Alder interactions, borate‐ester, disulfide bonds) or non‐covalent interactions (e.g. hydrogen bonds, *π*−*π* interactions, metal–ligand bonds, ionic bonds) [211]. These mechanisms can restore mechanical integrity and partially recover electrical function, thereby enhancing reliability and sustainability. However, achieving high healing efficiency and rapid healing kinetics while preserving sufficient semiconductor mobility remains challenging, and further materials design and mechanistic understanding are needed to enable self‐healing neuromorphic electronics [212]. Biodegradable materials, on the other hand, provide an attractive route for transient neuromorphic systems that can safely operate in physiological environments and naturally resorb after use [203]. Such materials undergo hydrolysis or oxidation to form biocompatible byproducts, eliminating the need for device retrieval and reducing long‐term environmental impact [213]. This transient behavior opens new possibilities for temporary implantable sensors, “green” wearable electronics, and disposable human‐machine interfaces [213, 214]. Biodegradability also provides inherent advantages for secure data disposal and minimizing electronic waste [213]. Nonetheless, biodegradable electronics are highly sensitive to moisture and must overcome stability and performance trade‐offs [203]. Continued advances in materials design, fabrication strategies, and control over degradation kinetics will be critical for developing biodegradable neuromorphic systems with capabilities approaching those of permanent devices, while also expanding the scope of neuromorphic bio‐interfaced applications they can enable [215].

At the device level, OECTs show particular promise for sensing and neuromorphic computing, yet several key performance metrics require improvement. Although the optimal specifications vary by application, switching speed, retention time, and environmental robustness of OECT‐based synapses frequently lag behind inorganic counterparts. In terms of speed, state‐of‐the‐art organic synaptic transistors have demonstrated sub‐microsecond write‐read cycling—approaching suggested targets for neuromorphic hardware [8, 40, 74, 83]. Nonetheless, emerging non‐volatile memory (eNVM) technologies now switch within a few nanoseconds [216, 217], and modern CMOS‐based DRAM operates at even higher frequencies [218, 219]. Because OECT operation fundamentally relies on ion‐mediated processes, speed is intrinsically constrained relative to purely electronic devices. The incomplete understanding of how material composition, microstructure, and ion–electron coupling dictate switching kinetics hinders rational device optimization [8, 40, 45, 220, 221]. Progress requires developing predictive, physics‐based models that explicitly related coupled ionic/electronic transport and redox kinetics to transient responses to guide materials selection and device design [220, 221]. Concurrently, exploring alternative switching mechanisms, emerging material systems (e.g. 2D polymers and 2D metal–organic frameworks (MOFs) [222, 223]), and advanced architectures (e.g., vertical stacks) may enable additional speed improvements. Device downscaling provides a promising route to further reduce switching times toward the nanosecond regime [38, 74], however, achieving this advancement necessitates simultaneous progress in device architecture and nanofabrication techniques [224].

Retention remains another bottleneck. Because OECT switching relies on ionic redistribution and volumetric redox reactions, its operation is fundamentally non‐equilibrium: internal electric fields and ionic gradients relax when the device is idle, and parasitic electrochemical reactions accelerate state loss [40]. Practical mitigations include tailored microstructure or partially crystalline channels for ion trapping, gated‐access or selector devices to limit parasitic currents, and advanced encapsulation schemes to slow environmental degradation [225]. These approaches have enabled OECT‐based synapses to reach retention times exceeding 10^4^ s [37, 74, 87], adequate for many online‐learning applications; [40] however, these values still fall short of the ∼10^6^‐second retention demonstrated by other eNVMs [216], the >6‐month stability required for long‐term bioelectronics [226], and the ∼10‐year target required for life‐long memory applications [6]. Moreover, many such remedies introduce additional circuit complexity or fabrication steps, increasing integration difficulty and cost. The electrochemical nature of OECT conductance modulation also reduces usable linear dynamic range and can force design trade‐offs that compromise state linearity or symmetry [40]. Developing quantitative models that explicitly link material and device parameters (e.g., mobility, volumetric capacitance, geometry) to key neuromorphic performance metrics (e.g. linear dynamic range, state symmetry, switching time) will be critical to facilitate further progress [227].

To provide a quantitative framework for assessing current progress and guiding future development, Table 3 compiles updated metrics for representative non‐volatile organic synaptic transistors and lists suggested target values aligned with the requirements of large‐scale, practical neuromorphic hardware.

**TABLE 3:** Comparison of key performance metrics for organic synaptic transistors and suggested target values.

Target metrics	State‐of‐the‐art organic synaptic transistor	Suggested values
Switching energy	∼80 fJ per write [83]	<1 pJ per event [8, 40]
Switching speed	∼1 µs per write‐read cycle [38, 74, 83]	<1 µs per event [8, 40]
LTP/LTD states	50 [38], 100 [83], 1000 [74]	∼100 [8, 40]
Dynamic range	∼2 [38, 83], 32 [74]	10∼20 [159, 228] depending on noise
Retention (demonstrated states)	∼600 s (11) [38, 225], 300 s (3) [83], 10^4^ s (6) [74]	10^3^∼10^8^ s [8, 40]
Switching noise (events)	∼2% (10^4^) [38], ∼2.4% (10^9^) [83], ∼0.49% (2000) [74]	<0.5% of weight range [8, 40]
Endurance (write‐read events)	10^8^ [38], 2 × 10^9^ [83]	∼10^9^ [6, 8, 40]
Size for integration	45 µm × 125 µm [38], 45 µm × 15 µm [83], 20 µm × 20 µm [74]	<1 µm^2^ [8, 40]
Fabricated array size	3 × 3 [38], 9 × 2 [74]	—

At the system level, miniaturization and scalability remain significant challenges. Photolithographic patterning organic materials is inherently difficult due to the chemical incompatibility between standard photoresists and conductive polymers [45, 229]. While progress has been made including the development of orthogonal photoresists and the demonstration of sub‐micrometer patterning for OSCs like PEDOT:PSS [230, 231], residual organic solvents can degrade device performance and introduce additional environmental risks [229, 232, 233]. Incorporating protective layers can mitigate direct exposure of organic semiconductors to harmful solvents, but this approach adds fabrication complexity and cost. To address the need for higher integration density, vertical device architecture offers a promising route to reducing the individual device footprint. Notable recent nanofabrication strategies have yielded high‐density vertical OECT arrays, achieving 10 µm × 10 µm devices (∼7.2 million OECTs cm^−^^2^) via direct electron‐beam patterning [234]. In parallel, a planar OTFT array fabricated using dual protective‐layer photolithography demonstrated 0.5 µm × 1.5 µm channels (∼5.1 million OTFTs cm^−^^2^), a density comparable to that of CMOS image‐sensor chips [232]. Crucially, however, aggressive device downscaling in these demonstrations often results in noticeable performance degradation, including significant reductions in mobility and transconductance [232, 234]. Emerging additive manufacturing (AM) technologies enable large‐scale, low‐cost fabrication of organic electronic systems, and fully printed, all‐solid‐state organic artificial synapse arrays have been reported (e.g. 5 × 9 [99]). However, printed synaptic transistors still lag photolithographically fabricated devices in performance, and AM has yet to achieve comparable OECT array densities.

Achieving highly uniform OSC films remains nontrivial due to their complex microstructures [161], and OECT performance is particularly sensitive to channel geometry because their operation relies on volumetric doping [154, 155, 235]. Stochastic variations arising during film drying or annealing can produce local differences in crystallinity, which in turn influence electronic conduction and lead to variations in transfer characteristics, mobility and volumetric capacitance [74, 236, 237]. These microscopic fluctuations propagate to threshold voltage, maximum transconductance, and ON/OFF ratio of organic devices [229, 232, 234], ultimately contributing to substantial device‐to‐device variability. Crystalline materials, such as small‐molecule semiconductors, have attracted interest as an alternative as their well‐defined molecular packing and narrow batch‐to‐batch variation enable more reproducible device performance [238, 239]. More recently, 2D MOFs have emerged as a promising class of nanoporous crystalline materials for achieving uniform OMIECs. Utilizing a layer‐by‐layer assembly strategy, an ultraflexible, large‐area OECT array with high device uniformity was demonstrated, highlighting the potential of MOF‐based electrochemical transistors (MOFECTs) for reliable, scalable integration [222, 240]. In parallel, AM technologies offer precise control over electrode composition, geometry, channel volume, topography, and electrolyte properties, and thus hold promise for improving device uniformity and reproducibility [241, 242]. However, consistent and uniform neuromorphic performance across large arrays has yet to be demonstrated.

As a result, demonstrations of more complex organic synaptic arrays remain limited in both miniaturization and array size, with representative examples including 45 µm × 125 µm (3 × 3 array) [38] and 20 µm × 20 µm (9 × 2 array) [74]. These dimensions are still far from the <1 µm^2^ device footprint suggested for practical neuromorphic integration [8, 40]. Moreover, current organic arrays remain orders of magnitude smaller than the scales achieved in state‐of‐the‐art neuromorphic computing systems based on CMOS—ranging from millions to billions of neurons [10] or compared to emerging hardware in‐memory computing arrays used in real applications (e.g. 128 × 64 [243], 32 × 32 [31]).

CMOS compatibility presents an additional set of challenges for large‐scale integration of organic neuromorphic electronic systems. Essential system functions, including compact nonlinear activation units and robust peripheral circuitry (e.g., selectors, drivers, readout and control electronics), have not yet been fully realized in purely organic form, which makes hybrid organic–CMOS architectures the most realistic short‐term path forward. However, this hybrid approach is hampered by a significant thermal mismatch: the electronic properties of many OSCs degrade at elevated temperatures (typically >150°C) due to phase transitions or morphology evolution [161], whereas back‐end‐of‐line (BEOL) CMOS processing commonly involves temperatures around ∼400°C. This thermal incompatibility severely restricts monolithic integration. Moreover, solution‐based processes, such as printing and coating, which are highly attractive for flexible, large‐scale, and low‐cost OECT fabrication with good device reproducibility [241], are generally incompatible with the high‐temperature requirements of standard BEOL processing, further complicating the integration of organic layers onto established CMOS platforms. Consequently, developing OSCs with enhanced thermal resilience is essential to bridge this gap, with recent work by Gumyusenge et al. demonstrating a notable step [244].

The lack of standardized measurement and reporting practices remains a major barrier to consistent and comprehensive comparison across the rapidly expanding field of OECT materials and device architectures [45, 220]. Despite recent proposals for unified characterization guidelines and proper device metrology, measurement practices remain heterogeneous, significantly hindering cross‐study comparisons [40, 220]. In neuromorphic studies, LTP/LTD characteristics are frequently reported together with read energy; however, the practical relevance is often obscured due to the lack of critical contextual information for the latter, particularly the number of stable non‐volatile states and the retention times required for neural inference. Meanwhile, write energy metrics, essential for assessing online‐learning capability and the true energy evaluation of LTP/LTD, are frequently omitted. Furthermore, the reporting of energy per switching event is inconsistent, with different articles presenting either the average or minimum switching energy, leading to confusion. Interpretation is further complicated when unstable transient states are mistakenly interpreted as non‐volatile memory states. Device‐to‐device variability is rarely quantified, limiting fair assessment across switching mechanisms, and retention time is sometimes reported for only a single conductance state, despite its typical state‐dependence [40]. Moreover, for reservoir computing OECTs, key figures such as memory capacity or the number of separable states are inconsistently reported, further impeding meaningful comparison with other material platforms [174]. Establishing community standards for materials characterization, device benchmarking, and reporting conventions is therefore crucial.

Algorithm–hardware mismatch represents an additional systematic constraint [45]. Conventional learning algorithms based on gradient descent and backpropagation assume high‐precision weights, abundant memory bandwidth and global access to network activity for propagating error signals and updating parameters—requirements that conflict with the locality, analog variability and limited memory of organic neuromorphic transistors. Accordingly, hardware‐aware algorithm development (local learning rules, reservoir and spike‐based schemes) and co‐design of devices and algorithms are necessary. Finally, while multimodal neuromorphic sensing is a highly promising application, integrating components that rely on disparate physical principles (optical, mechanical, chemical, biochemical) raises significant challenges in materials selection, process flow compatibility and interface standardization.

Beyond all‐organic neuromorphic electronics, hybrid organic–inorganic synaptic transistors—typically comprising an inorganic semiconductor channel coupled with an organic dielectric or electrolyte—represent a promising direction for next‐generation neuromorphic hardware [245]. These devices leverage the high mobility and stability of inorganic semiconductors together with the mechanical softness, ionic functionality, and biocompatibility of organic materials, enabling synaptic behavior through either field‐effect modulation or electrochemical doping [245]. Excellent synaptic performance has been reported in hybrids incorporating 2D inorganic channels with polymer electrolytes or organic ferroelectric layers [102, 104, 108, 246, 247]. Nonetheless, key challenges remain: organic electrolytes still suffer from limited ambient stability; processing incompatibilities between organic and inorganic components hinder scalable fabrication; and bandgap or surface‐energy mismatch at the hybrid interface can produce high‐density trap states that degrade synaptic performances and long‐term stability. Addressing these issues will be crucial for establishing hybrid organic–inorganic synapses as stable, high‐speed, low‐power, and scalable platforms for future neuromorphic integration.

In conclusion, organic neuromorphic electronics have progressed from isolated demonstrations of synaptic plasticity to increasingly sophisticated device–circuit prototypes that couple sensing, memory and processing in soft, bio‐integrable formats. Significant opportunities exist in wearable and bio‐interfaced applications where tunability, flexibility, stretchability and biocompatibility of organic materials confer clear advantages; however, persistent challenges in materials stability, device variability, and system‐scale integration must be addressed. Realizing practical technologies will therefore require sustained, interdisciplinary efforts that align materials chemistry, device physics, circuit engineering and learning‐algorithm design, together with community standards for benchmarking. With such coordinated work, organic neuromorphic hardware can mature into complementary technology that enables low‐power, soft, and biocompatible neuromorphic systems for applications inaccessible to rigid silicon platforms.

## Conflicts of Interest

The authors declare no conflicts of interest.

## Data Availability

The authors have nothing to report.
